# The genetic and epigenetic alterations in human hepatocellular carcinoma: a recent update

**DOI:** 10.1007/s13238-014-0065-9

**Published:** 2014-06-11

**Authors:** Ming Liu, Lingxi Jiang, Xin-Yuan Guan

**Affiliations:** Department of Clinical Oncology, University of Hong Kong, Hong Kong, China

**Keywords:** hepatocellular carcinoma (HCC), cancer hallmarks, genetic regulation, epigenetic regulation, therapeutic targets, HCC progression

## Abstract

Hepatocellular carcinoma (HCC) is one of the most frequent human malignancies worldwide with very poor prognosis. It is generally accepted that the progression of HCC is a long-term process with accumulation of multiple genetic and epigenetic alterations, which further lead to the activation of critical oncogenes or inactivation of tumor suppressor genes. HCC is characterized with multiple cancer hallmarks including their ability to proliferate, anti-apoptosis, invade, metastasis, as well as the emerging features such as stem cell properties and energy metabolic switch. The irreversible alterations at genetic level could be detected as early as in the pre-neoplastic stages and accumulate during cancer progression. Thus, they might account for the cancer initiating steps and further malignant transformation. In addition to genetic alterations, epigenetic alterations can affect the cancer transcriptome more extensively. Alterations in DNA methylation, histone modification, miRNAs, RNA editing, and lncRNAs might result in disrupted gene regulation networks and substantially contribute to HCC progression. In this review, the genetic and epigenetic alterations which significantly contribute to the malignant capabilities of HCC will be updated and summarized in detail. Further characterization of those critical molecular events might better elucidate the pathogenesis of HCC and provide novel therapeutic targets for treatment of this deadly disease.

## Introduction

Hepatocellular carcinoma (HCC) is one of the most frequent human malignancies worldwide. It is the sixth most prevalent cancer in the world and the third leading cause of cancer-related mortality (Parkin et al., 2005). The prevalence of HCC varies markedly in different regions. The highest incidence of HCC was found in Asia-pacific area (>20/100,000), while low incidence was found in Northern Europe and Northern America (<5/100,000) (Venook et al., 2010). China is a typical high-risk region which may account for more than 50% of HCC cases in the world (Yuen et al., 2009). The uneven distribution of HCC incidence among different geographic regions suggests that multiple genetic and environmental factors may interplay in the progression of this disease. Almost 70%–90% of HCC patients accompany with liver cirrhosis, which is believed to be the most important risk factor for HCC (Fattovich et al., 1997). Thus, all levels of viral infection, liver cytotoxicity, chronic inflammation which can lead to liver cirrhosis are important risk factors in the development of HCC (El-Serag and Rudolph, 2007).

It is widely accepted that carcinogenesis is a multi-step process with accumulation of genetic alterations in critical genes which regulate cell proliferation, growth, survival, apoptosis, adhesion, and metabolism (Vogelstein and Kinzler, 1993). The stepwise accumulation of genetic alterations in oncogenes and tumor suppressor genes will transform a normal cell and finally leads to carcinogenesis (Farber, 1984). The pathogenesis of HCC is also believed to be a long-term process which begins from the pre-malignant stage to the dysplastic stage and finally proceeds to the malignant stage (Thorgeirsson and Grisham, 2002). Better understanding of the genetic and epigenetic changes and their interactions at all the stages during HCC progression will greatly facilitate to elucidate the pathogenesis of HCC. In this review, we summarize the current knowledge of genetic and epigenetic alterations in the progression of HCC.

## Hallmarks of human hepatocellular carcinoma

**Figure 1:**
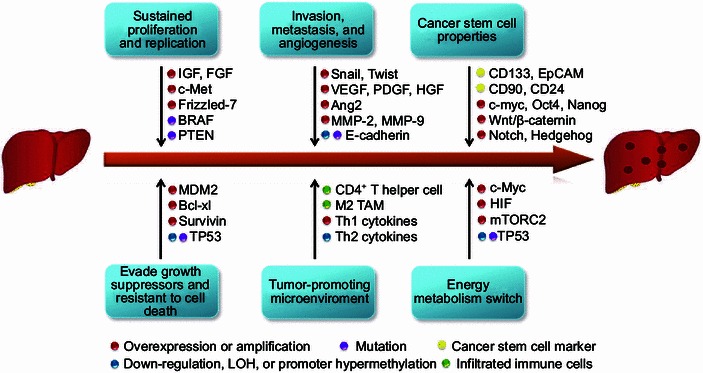
Multiple cancer hallmarks and the underlying molecular alterations in the progression of HCC. The progression of HCC is a multi-step process, which is characterized by several cancer hallmarks including sustained cell proliferation, evading growth suppressors, resistant to cell death, invasion, metastasis, angiogenesis, and deregulated energy metabolism. Multiple cellular and molecular alterations such as amplification or overexpression of oncogenes, hypermethylation or mutation of tumor suppressor genes, activation of cancer stem cells, and infiltration of immune cells, contribute to the malignant transformation of HCC

For decades, scientists are trying to unveil the underlied molecular mechanisms of cancer initiation and progression. However, the diverse characteristics and heterogeneity of cancer usually make confusion. Now, it is widely accepted that cancer evolves progressively from normal cells to malignant stages. During the multistep process, cancer cells acquired several hallmark capabilities which enable them to become tumorigenic and show all kinds of malignant phenotypes (Hanahan and Weinberg, 2000, 2011). Like other solid tumors, HCC is also characterized with those cancer hallmarks such as sustained cell proliferation, evading growth suppressors, resistant to cell death, invasion, metastasis, angiogenesis, and deregulated energy metabolism. The diverse malignant phenotypes of cancer cells usually associate with several genetic or epigenetic alterations of critical oncogenes or tumor suppressor genes. Thus, linking the hallmarks of HCC with genetic or epigenetic alterations will help to identify potential molecular mechanisms and find out novel targets for HCC treatment. Multiple cancer hallmarks and the underlying molecular alterations in the progression of HCC are summarized in Fig. 1.

### Sustained proliferation and replication

The growth and proliferation of normal cells are strictly regulated to maintain a homeostasis of cell number and tissue architecture. However, in tumors, cell growth and proliferation are usually deregulated, and sustained cell proliferation is one of the most common traits of cancer cells. Cancer cells have several ways to obtain the ability to proliferate and replicate rapidly. One mechanism is that they can autocrine or paracrine growth factors which will further activate the mitogenic signaling pathways (Lemmon and Schlessinger, 2010). In HCC, cell growth factors such as IGF, FGF were reported to be overexpressed in a way of autocrine or paracrine and further promote cell growth and proliferation (Kim et al., 1998; Yoshiji et al., 2002). Growth factor receptors are usually overexpressed or mutated in tumor cells, which results in persistent activation of downstream mitogenic signals. In HCC, the overexpression of HGF receptor c-Met can activate the downstream Ras/Raf/MEK signaling pathway (Ueki et al., 1997). Overexpression of the Frizzled-7 receptor leads to the activation of Wnt/beta-caternin signaling pathway in HCC (Merle et al., 2004). In addition to direct overexpressing growth factors and receptors, cancer cells can also activate the mitogenic pathways through affecting the upstream and downstream signal transducers or disruption of the negative feed back loop. For example, the oncogenic signal transducer BRAF is frequently mutated and consistently activated in HCC (Colombino et al., 2012); loss of function mutation of PTEN, which is the negative feed back regulator of PI3K/AKT pathway, was also frequently observed in HCC patients (Yao et al., 1999).

### Evading growth suppressors and resistant to cell death

In contrast to the proliferation stimulating signals, cells have also developed a growth inhibition system, which acts as a guardian of cell growth and proliferation. The guardian system is composed of series of growth and proliferation inhibitors, which are usually important tumor suppressor genes. The well-known tumor suppressor TP53 is at the center of the guardian system. Somatic mutation of TP53 is one of the most frequent genetic alterations in human cancer (Olivier et al., 2010). In HCC, TP53 was also found to be frequently mutated, and the common risk factors such as AFB1, HBV, and HCV are reported to cause TP53 mutation (Hussain et al., 2007; Ozturk, 1991). In addition to mutation of TP53 itself, the regulators of TP53 are usually found to be altered in cancer, such as MDM2. MDM2 ubiquitinates TP53 and promotes the proteasome-mediated degradation of TP53 (Kubbutat et al., 1997). Amplification and overexpression of MDM2 was frequently observed in HCC, and this might also account for the deregulated TP53 signaling pathway in HCC (Jablkowski et al., 2005). In addition to cell growth inhibition, cells can undergo apoptosis upon receiving extrinsic or intrinsic signals (Hengartner, 2000). The apoptotic signal is controlled by a group of counteracting pro- and anti-apoptotic proteins. The pro-apoptotic proteins such as Bax and Bak can enhance the permeability of mitochondria membranes and promote the release of cytochrome c, which further activates the caspase cascade. The anti-apoptotic proteins such as Bcl-2, Bcl-xl will counteract the pro-apoptotic proteins (Adams and Cory, 2007). Other regulatory proteins which interfere with the apoptotic signaling cascade, such as survivin, are also important anti-apoptotic components (Adams and Cory, 2007). The overexpression of anti-apoptotic proteins such as Bcl-xl and survivin are frequently observed in HCC patients (Shiraki et al., 2000; Takehara et al., 2001).

### Invasion, metastasis, and angiogenesis

Invasion and metastasis is one of the most common hallmarks of cancer, especially those with high grade malignancy. To gain invasive abilities, cancer cells usually undergo morphological changes termed “Epithelial-to-Mesechymal transition” (EMT). In epithelial tissues, cells are usually attached to each other or to the extracellular matrix (ECM) through adhesion molecules. However, in metastatic tumors, the adhesion molecules such as E-cadherin are usually down-regulated or mutated, and the loosened cell contact enables tumor cells to invade out from the primary niche (Cavallaro and Christofori, 2004). Conversely, adhesion molecules associated with cell migration such as N-cadherin, which are usually expressed in migrating cells, will be up-regulated in the aggressive cancer cells. The morphology of the cell will also change from the epithelial-like phenotype to fibroblastic mesenchymal-like phenotype (Voulgari and Pintzas, 2009). In HCC, altered expression of E-cadherin was frequently observed and correlated with clinical pathological features (Wei et al., 2002). Loss of heterozygosity (LOH) and CpG island hypermethylation have been proved to be the major mechanisms accounting for E-cadherin inactivation in HCC (Kanai et al., 1997). To date, several important transcriptional factors, such as Snail, Slug, Twist, and Zeb1/2, have been proved to be the key regulator of the EMT process. Overexpression of Snail and Twist has been closely correlated with HCC metastasis through inducing EMT (Lee et al., 2006; Sugimachi et al., 2003). In order to migrate from the original tissue, cancer cells need to degrade the barriers which hinder their movement, such as extracellular matrix. Matrix metalloproteinases (MMP) is a kind of secreted protease family, which can help digest the ECM (Stamenkovic, 2000). MMPs are synthesized in an inactive form, which could be activated after removing the pro-peptide domain (Pei et al., 2000). Overexpression and activation of MMPs are frequently observed in cancer cells, especially those with high metastatic ability (Rundhaug, 2003). Overexpression of MMPs, such as MMP-2 and MMP-9, are frequently observed in HCC patients and has been associated with cancer invasive potential (Arii et al., 1996; Giannelli et al., 2002).

Angiogenesis is another important feature of cancer. When tumor mass grows, the tumor cells need blood vesicles to provide enough nutrient and oxygen. Formation of tumor vessels can accelerate the proliferation, growth, and metastasis of cancer cells (Carmeliet and Jain, 2000). In the process of angiogenesis, tumor cells will secret several critical growth factors such as FGF, VEGF, which will activate the proliferation of endothelial cells or fibroblasts (Yancopoulos et al., 2000). VEGF has been proved to play a critical role in tumor angiogenesis including HCC. Overexpression of VEGF was correlated with HCC angiogenesis and vascular formation (Mise et al., 1996). Monoclonal antibodies targeting VEGF or small molecules inhibiting VEGF receptors have already being used in HCC treatment (Finn and Zhu, 2009). In addition to VEGF, other proangiogenic factors including platelet-derived growth factor (PDGF), hepatoctye growth factor (HGF), basic fibroblast growth factor (bFGF), and angiopoietin-2 (Ang2) are also elevated in the HCC plasma and make a substantial contribution to HCC angiogenesis (Semela and Dufour, 2004; Zhu et al., 2011).

### Tumor-promoting microenvironment

The initiation, growth, and metastasis of tumor not only depend on the malignant characteristics of cancer cells themselves, but also the tumor-promoting microenvironment (Joyce, 2005). Tumor grows in a complicated microenvironment, which is composed of stromal fibroblasts, endothelial cells, and infiltrating immune cells. These cells in the microenvironment can either secret growth factors to support tumor cell growth and angiogenesis, or produce pro-inflammatory cytokines and chemokines, which favors malignant transformation (Whiteside, 2008). For HCC, the tumor microenvironment might play a critical role in tumor initiation and progression. Etiological studies indicated that HCC mainly developed from liver cirrhosis, which is caused by chronic hepatitis virus infection, fatty liver disease, and alcohol abuse. The common trait during hepatocarcinogenesis is the sustained liver damage and regeneration, which leads to an inflammatory microenvironment in the liver. The inflammatory microenvironment supports the recruitment and activation of hepatic stellate cells and macrophages, which further produce components of the ECM, growth factors, and chemokines for angiogenesis and fibrosis (Hernandez-Gea et al., 2013).

There are several kinds of cells closely associated with HCC tumor microenvironment. Like other solid tumors, the most common cells observed in the tumor microenvironment are immune cells. In response to inflammatory signal, immune cells including T cells, B cells, macrophages, and dendritic cells will infiltrate into the tumor mass, and produce several kinds of cytokines, which either inhibit or promote tumor growth (Hernandez-Gea et al., 2013). The most common tumor-infiltrating lymphocyte is CD^4+^ T helper cells. The cytokines secreted by Th cells could further be divided into two groups including Th1-like cytokines and Th2-like cytokines. A unique signature of increased Th1 cytokines (IL-1, IL-2, TNFα, etc.) but decreased Th2 cytokines (IL-4, IL-8, IL-10, etc.) was frequently observed in HCC tumor microenvironment and associated with poor prognosis of HCC patients (Ye et al., 2003). Tumor associated macrophage (TAM) is another important subset of infiltrated immune cells in the tumor microenvironment. High density of infiltrated TAMs usually associated with poor prognosis of HCC patients (Ding et al., 2009). TAMs can either secret tumor-promoting growth factor, cytokines, chemokines, etc. to facilitate tumor growth, or suppress the anti-tumor immunity in HCC tissues. The macrophages can also be divided into two subgroups like T helper cells. TAMs resemble the M2 macrophages, which provide the formation of Th2 tumor microenvironment (Bingle et al., 2002).

### Cancer stem cell properties

According to the cancer stem cell (CSC) model, cancer originates from a subset of stem like cells that have self-renewal properties. Malignant cancer cells usually have similar properties as embryonic cells characterized with elevated stemness markers and maintained in a dedifferentiated status (Reya et al., 2001). Assessing the differentiation level of tumor is often conducted in the clinic, and the poorly-differentiated tumors are closely associated with patient prognosis. The histologically poorly-differentiated tumors usually show an embryonic-like gene expression signature (Ben-Porath et al., 2008). In HCC, certain cell populations have been identified as potential cancer stem cells. The “Oval cells” which give rise to hepatoblast cells and primitive bile duct cells during liver development are considered to be origins of liver cancer stem cells. Oval cells express cellular markers of both hepatocytes and bile duct, and have the potential to differentiate into both lineages. Therefore, oval cells are considered to be liver progenitor cells, which might be initiating cells in hepatocarcinogenesis (Mishra et al., 2009). A small group of cells known as “side population” (SP), which are able to pump out nucleus dye via ABCG2-transporters, are also considered to be potential liver cancer stem cells. SP cells have enhanced self-renewal ability *in vitro* and tumorigenic ability *in vivo*. Molecular characterization of SP cells indicated that several oncogenic signaling pathways associated with cancer self-renewal and differentiation, are activated in SP cells (Marquardt et al., 2011). In addition to oval cells and SP cells, several cell surface markers have been identified as cancer stem cell markers, including CD133, EpCam, CD90, CD24, etc. The isolated cancer cells using those markers all shown strong self-renewal properties and tumorigenic ability with only few cells injected in xenograph mouse model (Lee et al., 2011; Ma et al. 2007; Yamashita et al., 2009; Yang et al., 2008)

Several oncogenic signaling pathways have been proved to play important roles in regulating cancer stemness and differentiation. The well-known oncogene c-myc was reported to account for the embryonic stem cell like phenotype of cancer cells (Kim et al., 2010). Inactivation of the myc network can induce the differentiation of HCC cells (Shachaf et al., 2004). Wnt/β-caternin is another important signaling pathway in regulating cancer stemness and differentiation. The secreted wnt will inhibit the cytoplasmic degradation of β-caternin, which further activates the β-caternin/TCF transcriptional machinery and promotes the transcription of several stemness markers such as Epcam, Ck19, and CD44, etc. (Fodde and Brabletz, 2007; Yamashita et al., 2007). In addition, other critical signaling pathways involved in regulating stem cell self-renewal and differentiation, such as Oct4, Nanog, Sox2, and Notch/Hedgehog have been reported to be important in maintaining the pluripotency of liver cancer progenitor cells and are frequently activated in HCC (Patil et al., 2006; Yuan et al., 2010).

### Energy metabolism switch

Altered energy metabolism switch from oxidative phosphorylation to glycolysis, known as Warburg effect, has been widely accepted as an emerging hallmark of cancer. Early in the 1930s, oncologists and scientists have noticed the alteration of energy metabolism in malignant tumors. Glycolysis was preferentially used as the main program for energy metabolism in tumor cells even in the presence of oxygen (Cairns et al., 2011). In normal cells, ATP is mainly generated from the tricarboxylic acid (TCA) cycle, followed by oxidative phosphorylation in the mitochondria. Oxidative phosphorylation generates 36 molecules of ATP from one molecule of glucose. In contrast, the glycolysis only generates two molecules of ATP from one molecule of glucose (Kroemer and Pouyssegur, 2008). Although glycolysis is not efficient in generating energy, it can provide a large amount of nucleotides, fatty acids, membrane lipids to support the synthesis of macromolecules, which are required for rapid tumor growth. In compensation, cancer cells increased the glucose intake by up-regulating glucose transporters and enhancing the usage of glutamine. The Warburg-like metabolic switch found to be present in many rapidly dividing embryonic tissues further supported the hypothesis that glycolysis could generate diverse intermediates for biosynthetic programs that are important for active cell proliferation (Hsu and Sabatini, 2008).

The reduced dependence of cancer cells on oxidative phosphorylation is not only due to defects in the cellular components of TCA cycle, but also strictly regulated by series of oncogenes and tumor suppressor genes. Oncogenic activation of c-myc, Ras, Akt, and HIF, or inactivation of tumor suppressors such as TP53 can drive metabolism changes in cancer cells (Levine and Puzio-Kuter, 2010). Like other solid tumors, metabolic remodeling from mitochondrial oxidation to aerobic glycolysis is common in human HCC (Beyoglu et al., 2013). c-Myc was reported to induce mouse liver tumors with elevated glucose and glutamine catabolism (Yuneva et al., 2012). Hepatic mTORC2 can activate glycolysis and lipogenesis through phosphorylating AKT (Hagiwara et al., 2012). Multi-kinase inhibitor such as sorafenib, which targets those oncogenic signaling pathways, was reported to be able to reverse the metabolic reprogramming in HCC (Fiume et al., 2011).

## Genetic alterations in HCC

Genetic alteration is one of the most important mechanisms associated with HCC initiation and progression. Genetic changes could be observed as early as in the pre-neoplastic lesions of cirrhotic liver, and it is thought to be the initiating events in hepatocarcinogenesis. The irreversible genetic abnormalities accumulate in hepatocytes, which further cause disrupted gene expression, and finally lead to malignant transformation. Genetic alterations could be divided into several types, including large chromosomal amplification, translocation, deletion, small fraction loss, and single nucleotide variation. The genetic changes at all levels usually result in the activation or loss-of-function of certain important oncogenes or tumor suppressor genes, which govern cell growth and proliferation.

### Chromosomal instability

Chromosomal instability is the most common genetic changes in HCC. It could be induced by either error during mitosis or disruption in DNA replication and repair. The chromosome abnormalities could be observed as the gain and loss of whole chromosome arms, or just amplification and deletion of small chromosomal fragments. According to the comparative genomic hybridization (CGH) data, chromosome 1q and 8q are frequently amplified, while chromosome 1p, 4q, 6q, 9p, 16p, 16q, and 17p are frequently lost in HCC (Guan et al., 2000). The observation of chromosomal alterations in preneoplastic liver tissues indicated that chromosome instability may occur in the early stage of HCC, and accumulates during tumor progression (Kondo et al., 2000). Thus chromosome instability may activate certain cancer driver genes during hepatocarcinogenesis.

Amplification of chromosome 1q is one of the most frequently observed chromosome abnormalities in HCC. The minimal region of 1q21 was found to be amplified in more than 50% of HCC patients. A well characterized oncogene CHD1L is localized in that region (Ma et al., 2008). CHD1L was found to have several oncogenic roles such as inhibiting cell apoptosis, regulating cell mitosis, and promoting cell epithelial-to-mesenchymal (EMT) transition during hepatocarcinogenesis (Chan et al., 2012; Chen et al., 2010; Chen et al., 2009a). In a transgenic mice model, CHD1L could induce spontaneous liver tumors formation (Chen et al., 2009b). In addition, CHD1L could regulate p53 stability, potentially via interacting with SCYLIBP1, which modulates the pirh2-mediated ubiquitin degradation of p53 (Hu et al., 2012). Adenovirus-mediated silencing of CHD1L could inhibit HCC tumorigenesis in xenograft mouse model further suggested CHD1L as a potential therapeutic target in HCC treatment (Chen et al., 2011). In addition to chromosome 1q21, a recent study indicated that a novel potential oncogene Maelstrom (MAEL) at 1q24, could induce EMT and enhance stemness properties of HCC cells (Liu et al., 2013). Chromosome 8q is another highly amplified chromosome arm in HCC, especially at the 8q24 region (Wang et al., 2002). Well-known oncogenes including *c-Myc* and *PTK2* are located at this region, and have been characterized for their oncogenic effects on HCC development (Okamoto et al., 2003; Santoni-Rugiu et al., 1998). In addition to 8q24, the chromosomal region proximal to the centromere is also frequently amplified in HCC (Parada et al., 1998). A serine/threonine kinase SGK3, which shares great similarity with AKT, was found to be frequently amplified and confer AKT-independent oncogenic roles in HCC (Liu et al., 2012).

Chromosome segmental loss is also frequently observed in HCC. The minimal region of 1p35-36 was found to be deleted in more than 50% HCC patients. Several tumor suppressors such as 14-3-3 σ and Rb-interacting zinc finger 1 (RIZ1) were located in this region (Iwata et al., 2000). Loss of the short arm of chromosome 8 has been recurrently observed in HCC. A minimal region of 8p21-22 was found to be frequently deleted in HCC. Deleted in liver cancer 1 (DLC-1), which is a homolog of the rat RhoGAP gene, is located in that region (Yuan et al., 1998). DLC-1 is frequently deleted in HCC tissues due to allele loss and promoter hypermethylation (Wong et al., 2003). Restore DLC-1 expression in hepatoma cells could induce cell apoptosis, and inhibit tumor growth (Zhou et al., 2004). Chromosome 16q is another region with frequent deletion in HCC. Cell adhesion molecule E-cadeherin (CDH1), which inhibits cell proliferation and metastasis, is located on 16q22 (Kanai et al., 1997). Another tumor suppressor gene Tyrosine aminotransferase (TAT), which might contribute to the pathogenesis of HCC, is also located on 16q22 (Fu et al., 2010). Recently, a significant allele-specific imbalance was identified in the 16q23 region in a cohort of HCC patient due to LOH. The affected gene *Oxidative Stress-Induced Growth Inhibitor 1 (OSGIN1)* can directly induce cell apoptosis in HCC cells and contributes significantly to the progression of HCC (Liu et al., 2014). The well-known tumor suppressor TP53 is mapped to 17p13.1, which is also a recurrently lost region in HCC. The 17p13 region was characterized with DNA hypermethylation, and loss of 17p13.1 was closely associated with TP53 mutation (Nishida et al., 1993). Summary of chromosome alterations and candidate target genes reported in HCC was listed in Table 1.

**Table 1:** Aberration of chromosome and candidate target genes reported in HCC.

Chromosome	Type of aberration	Frequent aberration region and candidate target genes (Location)	References
1q	Gain	*CKS1B* (1q21.2), *CHD1L* (1q21.1), *JTB* (1q21), *MDM4* (1q32.1)	Chen et al., 2010; Kim et al., 2008
1p	Loss	*p18* (1p32)*, 14-3-3σ* (1p35), *p73* (1p36.3), *RIZ* (1p36.13-p36.23)	Nishimura et al., 2005; Iwata et al., 2000; Fang et al., 2000
3q	Gain	*Gankyrin* (3q28)	Higashitsuji et al., 2000
3p	LOH, CpG methylation	*RASSF1A* (3p21.3), *CTNNB1* (3p21), *TGF-1βR11* (3q22)	Zhang et al., 2002; Miyoshi et al., 1998
4q	LOH		Zondervan et al., 2000
6p	Gain		Chochi et al., 2009
6q	LOH	*M6P/IGF2R* (6q26-q27)	Oka et al., 2002
8q	Gain	*c-Myc* (8q24.21), *PTK2* (8q24.3), *EIF3S3* (8q23.3), *SGK3* (8q13.1)	Santoni-Rugiu et al., 1998; Okamoto et al., 2003; Liu et al., 2012
8p	LOH, CpG methylation	*DLC-1* (8p21.3-22), *LPTS* (8p23), *CSMD1* (8p23.2)	Yuan et al., 1998
9p	LOH, CpG methylation	*CDKN2A* (9p21), *CDKN2B* (9q21),	Wang et al., 2000
10q	LOH	*PTEN*/*MMAC1* (10q23.3)	Fujiwara et al., 2000
11q	Gain	*cyclinD1* (11q13)	Nishida et al., 1994
11p	LOH, CpG methylation	*KAI1* (11p11.2)*, IGF-2* (11p15)*, TSLC1* (11q23.2)	Tsujiuchi et al., 2007
13q	LOH	*Rb1* (13q14.2)*, BRCA2* (13q12.3)*, Tg737* (13q12.1)*, TFDP1* (13q34)*, CUL4A* (13q34)*, CDC1* (13q34)	Kuroki et al., 1995; Yasui et al., 2002
16q	LOH, CpG methylation	*CDH1* (16q22.1)	Wang et al., 2000
16p	CpG methylation	*Axin1* (16p13.3), *SOCS-*1 (16p13.3)	Li et al., 2013c; Ko et al., 2008
17p	LOH	*p53* (17p13.1), *HIC-1* (17p13.3), *HCCS1* (17p13.3)	Nishida et al., 1993; Kanai et al., 1999; Zhao et al., 2001

### Genomic mutations

In addition to large chromosomal alterations, genomic mutation is another important genetic alteration which contributes to tumor initiation and progression. Genomic mutations could be divided into germline mutations and somatic mutations. A germline mutation is usually inherited, and exists in all cell types of a body. Germline mutations are rare, and usually account for cancer risk in certain families. In contrast, somatic mutations usually exist in tumor tissues or preneuplastic tissues, and accumulate during cancer progression. Somatic mutations are more common, and might account for malignant transformation of sporadic tumors. Missense genomic mutations at the open reading frame can either lead to loss-of-function of tumor suppressors or gain-of-function of oncogenes. In addition, mutations at the non-coding region of the genome can also affect cancer risk and progression, for they may change the transcription, translation, or stability of the gene product.

With the development of the next-generation high throughput deep sequencing technology, scientists now are able to identify somatic mutation patterns in a certain tumor tissue, like HCC. Recently, two groups have sequenced the whole genome of several HCC tumor tissues and their paired non-tumor tissues (Fujimoto et al., 2012; Guichard et al., 2012). Recurrent somatic mutations were enriched in several signaling pathways including wnt/β-catenin, p53/cell cycle control, chromatin remodeling, PI3K/Ras signaling, and oxidative and endoplasmic reticulum stress. The wnt signaling pathway was found to be the most frequently altered in HCC. Activating mutation in CTNNB1 was found in 32.8% of HCC patients. While the inactivating mutations of AXIN1 and APC was found in 15.2% and 1.6% of HCC patients, respectively. The second most altered pathway in HCC is the p53 signaling pathway. Inactivation mutation of p53 was present in 20.8% of HCC patients and mutation in CDKN2A was identified in 8% of HCC patients. In addition to other traditional signaling pathways, which are frequently mutated in cancer, several recent studies reported that the components of the chromosome remodeling complex are frequently mutated in many cancer types including HCC. The mutation of the SWI/SNF chromatin remodeling complex component ARID1A was detected in more than 20% of HCC patients. These indicated that the chromatin remodeling complex might play important roles in cancer initiation and progression.

### Cancer susceptibility genes

It is widely accepted that genetic polymorphisms at cancer susceptibility genes can affect the cancer risk of certain population. Unlike genetic mutations, which directly cause loss-of-function or gain-of-function of gene products, and usually affect important oncogenes or tumor suppressor genes involved in critical signaling pathways, nucleotide changes in cancer susceptibility alleles might not directly cause dramatic functional changes of a protein. Instead, cancer susceptible genetic variations might slightly affect the function of a protein, for example the efficiency of an enzyme, thus confer an increased cancer risk for certain population. A wide range of genes are associated with cancer risk, including carcinogen metabolism genes, anti-tumor immune response genes, and genes associated with cellular response to stress (Antoniou et al., 2010).

Genome-wide association study (GWAS) is emerging as a powerful tool to identify cancer susceptibility alleles in tumorigenesis. GWAS examines common genetic variants in different individuals and identifies variants associated with certain disease. In contrast to mendelian linkage analysis, which aims to identify highly penetrant tumorigenic mutations, GWAS is powerful in identifying less penetrant tumor susceptibility alleles, which are more common and might be important in cancer initiation and progression. Several GWASs have been performed to identify susceptibility alleles associated with HCC. Intronic SNP (rs17401966) in KIF1B on chromosome 1p36.22 has been linked to HBV-associated HCC (Zhang et al., 2010). Chromosome loci 6p21.32 and 21q21.3 have also been associated with HCC in chronic HBV carriers (Li et al., 2012). A recent study indicated that genetic variations in STAT4 and HLA-DQ genes may confer risk of HBV-related HCC (Jiang et al., 2013). SNP (rs2596542) in the 5′ flanking region of MICA on 6p21.33 has been linked to HCV-associated HCC (Kumar et al., 2011).

### MicroRNAs (miRNAs)

MicroRNA, a class of non-coding RNAs, has been identified as important regulators of gene expression at post transcriptional levels. Emerging evidences indicated that miRNAs are associated with the development and progression of HCC. In recent years, intensive investigations have been conducted to find out the abnormally expressed miRNAs and their roles in HCC development and progression. Some miRNAs can regulate the proliferation pathways via modulating cyclins or cyclin-dependent kinases, such as miR-122a and miR-221 (Gramantieri et al., 2007). Some miRNAs can help HCC cells to escape from apoptosis by targeting pro-apoptotic protein. For example, Bmf, a proapoptotic protein, is a target of miR-221 (Gramantieri et al., 2009). On the contrary, other miRNAs can promote HCC apoptosis. For example, the anti-apoptotic proteins Bcl-2 and Mcl-1 are two direct targets of miR-29 (Xiong et al., 2010). As two of the most critical hallmarks of HCC, invasion and metastasis are also regulated by miRNAs. On the one hand, the pro-metastatic miRNAs can promote cell migration and spreading in HCC. For example, miR-106b can promote HCC cell migration and invasion by activating epithelial-mesenchymal transition (EMT) process (Yau et al., 2013). On the other hand, several miRNAs such as let-7g, miR-139, and miR-195 can suppress metastasis and progression of HCC (Ji et al., 2010; Wang et al., 2013b). Additionally, some miRNAs have been reported to enhance the ability of self-renewal and tumorigenicity of HCC. MiR-130b can regulate CD133(+) liver cancer stem cells via silencing TP53INP1 (Ma et al., 2010). Inhibition of miR-181 can result in a reduction in EpCAM(+) HCC cell quantity. Exogenous miR-181 expression in HCC cells led to an enrichment of EpCAM(+) HCC cells and promote tumor initiating ability (Ji et al., 2009). Summary of the abnormally expressed miRNAs and their functions are listed in Table 2.

**Table 2:** MiRNAs aberrantly expressed and validated target genes in hepatocellular carcinoma.

miRNA	Expression	Gene targets	Function	References
miR-17-5p	Up	p38, MAPK pathway, E2F-1, c-MYC	Promote tumor growth and metastasis.	Yang et al., 2010; El Tayebi et al., 2013
miR-18a	Up	ERα	Promote proliferation.	Liu et al., 2009
miR-18b	Up	TNRC6B	Promote cell proliferation and loss of cell adhesion.	Murakami et al., 2013
miR-21	Up	PTEN, RECK, PDCD4	Inhibit apoptosis, promote cell migration and invasion.	Meng et al., 2007; Zhou et al., 2013
miR-106b	Up	E2F1, RhoGTPases, RhoA, RhoC	Promote cell migration and stress fiber formation.	Yau et al., 2013
miR-130b	Up	TP53INP1	Promote CD133(+) liver cancer stem cell growth and self-renewal.	Ma et al., 2010
miR-143	Up	FNDC3B	Promote tumor metastasis.	Zhang et al., 2009
miR-151	Up	RhoGDIA, FAK,	Stimulate tumor invasion and metastasis.	Ding et al., 2010; Luedde, 2010
miR-181b	Up	TIMP3	Promote tumor metastasis.	Wang et al., 2010a
miR-181	Up	CDX2, GATA6,NLK	Promote EpCAM(+) liver cancer stem cell growth and self-renewal.	Ji et al., 2009
miR-185	Up	KCNN3	Association with HCC venous metastasis.	Budhu et al., 2008
miR-210	Up	VMP1	Promote hypoxia-induced HCC cell metastasis.	Ying et al., 2011
miR-221/222	Up	CDKN1B/p27,CDKN1C/p57, DDIT4, PTEN, Bmf, TIMP3, PPP2R2A	Inhibit apoptosis, promote tumor growth and metastasis.	Fornari et al., 2008; Gramantieri et al., 2009
miR-224	Up	API-5, CDC42, CDH1, PAK2, BCL-2, MAPK1, PPP2R1B.	Promote cell proliferation, migration, invasion, and inhibit cell apoptosis.	Wang et al., 2008; Zhang et al., 2013
miR-1	Down	FoxP1, MET, HDAC4.	Inhibition of cell growth and reduced replication potential.	Datta et al., 2008
let-7	Down	c-Myc, p16, Bcl-xl, COLIA2.	Inhibition of cell growth and proliferation.	Wang et al., 2010b; Ji et al., 2010
miR-26a	Down	Cyclin D2, Cyclin E2,Cyclin E1, CDK6, IL-6	Inhibit tumor growth, metastasis, and invasion.	Yang et al., 2013
miR-29	Down	MEG3, Bcl-2, Mcl-1	Promotion of apoptosis and inhibition of tumor growth	Xiong et al., 2010
miR-34a	Down	c-Met	Inhibition of cell growth, migration, and invasion.	Li et al., 2009
miR-122	Down	CyclinG1, ADAM10, SRF, IGF1R, PTTG1, PBF,CUTL1, NDRG3, MDR-1	Inhibit viral replication and cell proliferation.	Song et al., 2012; Li et al., 2013a; Xu et al., 2010; Gramantieri et al., 2007
miR-124	Down	ROCK2, EZH2, PIK3CA	Inhibit tumor growth, invasion, and metastatic potential of HCC.	Zheng et al., 2012; Lang and Ling, 2012
miR-126	Down	ROCK2, c-Fos	Inhibit cell invasion and migration.	Wong et al., 2011
miR-145	Down	OCT4, IRS1, IRS2, IGF signaling, HDAC2.	Inhibit cell proliferation, migration, and invasion.	Wang et al., 2013a; Law et al., 2012; Noh et al., 2013
miR-148a	Down	HPIP, AKT/ERK/FOXO4/ATF5 pathway	Inhibit tumorigenesis.	Xu et al., 2013
miR-195	Down	cyclin D1, CDK6, E2F3, LATS2, VEGF, VAV2, CDC42, IKKα and TAB3, TNF-α/NF-κB pathway	Inhibit G1/S transition, angiogenesis, and metastasis, promote apoptosis.	Xu et al., 2009; Wang et al., 2013b; Ding et al., 2013
miR-199a-3p	Down	mTOR, c-Met, CD44	Inhibit cell growth and metastasis	Fornari et al., 2010; Henry et al., 2010
miR-214	Down	XBP-1, HDGF, EZH2, CTNNB1, β-catenin signaling pathway	Inhibit cell proliferation, promote cell apoptosis, and suppress tumor vascularity.	Shih et al., 2012; Xia et al., 2012
miR-223	Down	stathmin1	Inhibit cell proliferation	Wong et al., 2008
miR-375	Down	YAP, AEG-1, ATG7	Inhibit tumorigenesis	Liu et al., 2010; He et al., 2012; Chang et al., 2012

### RNA editing

The RNA transcripts are usually faithfully transcribed from the genome without sequential changes after RNA processing. However RNA editing is a molecular process which could result in nucleotide changes at specific sites of the RNA transcripts. Thus, RNA editing could add great diversity to the posttranscriptional regulation of gene expression (Gott and Emeson, 2000). RNA editing can modify the transcribed RNA sequences via nucleotide insertion, deletion, and substitution. The most common type of RNA editing in human is the adenosine to inosine (A-to-I editing), which is mediated by theadenosine deaminase acting on dsRNA (ADAR) family of enzymes. The A-to-I editing can affect various types of RNA molecules including mRNAs, microRNAs, viral RNAs, etc. (Athanasiadis et al., 2004). RNA editing at the coding regions of the transcripts may lead to non-synonymous amino acid changes in the gene products, which might affect the biological functions of the proteins. Recent studies have linked A-to-I RNA editing to hepatocarcinogenesis. Through next-generation RNA sequencing technology, an A-to-I editing event within the AZIN1 transcript was identified in the tumor tissues from HCC patients. Hyper-editing of AZIN1 transcripts in the tumor cells resulted in a recording of AZIN1 protein from Serine to Glysine at coden 367. The edited AZIN1 showed strong oncogenic phenotypes on HCC cell lines and mouse models, compared with the wild type form. The frequency of RNA editing in the tumor tissues also significantly associated with the prognosis of HCC patients (Chen et al., 2013). The disrupted RNA editing was found to be mediated by differential expression of ADARs in HCC (Chan et al., 2013). Further characterization of the RNA editing events in HCC might help elucidate the pathogenesis of this disease (Li et al., 2013b).

## Epigenetic alterations in HCC

Genetic alterations are irreversible changes that affect the DNA sequence of the genome. In contrast, epigenetic regulations do not change the sequence of the genome but affect the chromatin structure and gene transcription. Epigenetic regulations affect gene products at multiple levels, including both transcriptional level and post-transcriptional regulation, which added great diversity to the gene regulation network. DNA methylation, histone modification, and recently emerging lncRNA, are major forms of epigenetic regulations. Alterations at cellular machineries governing those processes are frequently observed in cancer cells including HCC. The epigenetic alterations usually result in the activation of oncogenes or inactivation of tumor suppressor genes, which further contribute to malignant cancer hallmarks. Increasing evidences suggested that epigenetic alterations are evolving as an important mechanism in cancer initiation and progression (Momparler, 2003).

### DNA methylation

In a normal cell, DNA methylation and demethylation is an important mechanism in regulating gene expression and chromatin structure. DNA methylase (DNMT) catalyze the methylation of cytosine at CpG islands at the promoter region of a gene. However, in tumor cells, the promoter methylation pattern is usually changed. Aberrant DNA methylation at the promoter region is an important mechanism of tumor suppressor gene inactivation. The hypermethylated CpG islands at the promoter region will prevent the binding of RNA polymerase and transcriptional factors, thus inhibit the transcription of the target genes. In addition, the hypermethylated protein will recruit m^5^CpG-binding domain (MBD) containing proteins, which will be an obstacle for the binding of transcriptional factors to the promoters, thus inhibit gene transcription (Hendrich and Bird, 1998).

In HCC, CpG island hypermethylation is frequently observed at the promoter region of important tumor suppressor genes. Suppressor of cytokine signaling (SOCS-1), which regulates the JAK/STAT signaling pathway, was found to be silenced in more than 60% of HCC patients due to promoter hypermethylation (Yoshikawa et al., 2001). The well-known tumor suppressor APC and E-cadeherin were also hypermethylated in 53% and 49% of HCC patients, respectively (Yang et al., 2003). Methylation profiling of multi-step HCC tumors revealed that the number of genes methylated showed stepwise increase with the progression of cancer stage. The observation of tumor suppressor gene hypermethylation in the para-tumor liver tissues and cirrhotic livers indicated that aberrant promoter methylation occurs in the early stage of hepatocarcinogenesis and increased progressively during cancer progression (Lee et al., 2003). In addition, genome-wide DNA methylation analysis revealed that epigenetic silencing of multiple tumor suppresors in HCC could result in the activation of several oncogenic signaling pathways including Ras, JAK/STAT, and Wnt/β-catenin (Calvisi et al., 2007).

There are several proposed hypotheses for the aberrant DNA methylation in cancer. One possible mechanism is the aberrant expression of DNMT1. As part of the DNA replication complex, DNMT1 transfer the methyl to the DNA immediately after DNA replication. In cancer cells, DNMT1 is usually abnormally expressed, and this will commit methylation errors during DNA replication (Vertino et al., 2002). Significant increase of DNMT1 was observed in HCC patients (Saito et al., 2003). In addition to DNMT1, which mainly accounts for the maintenance of methylation pattern of the genome, other DNMT family members such as DNMT3A and DNMT3B can directly add methyl groups to unmethylated DNA. DNMT3A and DNMT3B are responsible for novel methylation pattern formation in the genome (Okano et al., 1999). DNMT3A and DNMT3B were reported to be associated with hypermethylation of several important tumor suppressor genes, such as CDKN2A, CDKN2B, CDH1, and Rb1 (Mizuno et al., 2001). The expression of DNMT3A and DNMT3B are both significantly overexpressed in HCC compared with the non-cancerous liver tissues (Oh et al., 2007).

### Histone modification and chromatin remodeling

Chromatin is the fundamental structure of the genome, which is constituted by nucleosome particles. The chromatin structure is important to gene transcription. In active transcription sites, the chromatin will be loosened, so that the DNA can be exposed to transcriptional factors for transcription initiation. This open chromatin structure is termed “euchromatin”. In contrary, some of the chromatin structure is heavily condensed and the transcriptions of those genes within those regions are inhibited. The condensed chromatin structure is termed “heterochromatin”. Thus, chromatin structure is of critical importance in regulating gene expression in a temporal and spatial dependent manner (Wang et al., 2007a).

Histone modification is playing a central role in chromatin structure regulation. Covalent modification of histones with methylation or acetylation will result in the chromatin structural change and could be used as markers for chromatin structure. There are two histone modification markers which represent an active transcription. Trimethylation of H3 lysine 4 (H3K4Me3) is often observed at the promoter region of actively transcribed genes. Trimethylation of H3 lysine 36 (H3K36Me3) is also closely associated with active transcription. In contrary, trimethylation of H3 lysine 27 (H3K27Me3) and trimethylation of H3 lysine 9 (H3K9Me3) are associated with repressed transcription (Kouzarides, 2007). It is recognized that histone modification is catalyzed by several enzymes which modulate the histone markers. The histone modifiers include histone methyltransferases (HMT), histone acetylatransferase (HAT), and histone deacertylase (HDAC), etc. Abnormal expression of those histone modifiers which further drives epigenetic alterations are frequently observed in cancer cells. In HCC, overexpression of EZH2, which is the histone methyltransferase for H3K27Me3, has been proven to contribute to the malignant transformation and poor prognosis of HCC (Chen et al., 2007; Sudo et al., 2005). The P300/CBP-associated factor (PCAF), which is a well-known HAT, was expressed at low level in HCC, and has been proven to inhibit HCC tumorigenesis both *in vitro* and *in vivo* (Zheng et al., 2013). HDAC inhibitors have been suggested to specifically induce apoptosis in hepatoma cells but not in primary hepatocytes. And these results greatly supported the potential application of HDAC inhibitors in clinical treatment of HCC patients (Armeanu et al., 2005; Pathil et al., 2006).

In addition to histone modifiers, the ATP-dependent chromatin remodeling complex, which utilize ATP to mobilize nucleosomes along DNA, are also closely involved in tumorigenesis. The ATP-dependent chromatin remodeling family could be further divided into four subfamilies including: the SWI/SNF (Switching defective/, sucrose non-fermenting) family, the ISWI family (imitation SWI), the NuRD/CHD (Nucleosome remodeling and deacetylation/Chromodomain helicase, DNA binding) family, and the INO80 (inositol requiring 80) family (Wang et al., 2007). Whole-genome sequencing has identified recurrent somatic mutations in genes associated chromatin remodeling complex, including ARID1A, ARID2, and SMARCA4 (Guichard et al., 2012; Li et al., 2011). The frequently observed inactivating mutations indicated the important roles of chromatin remodeling complex in HCC development. The ATPase and putative DNA helicase RuvB-like 2 (RUVBL2) was found to be overexpressed in HCC and has contributed to the malignant transformation (Rousseau et al., 2007). Copy number loss or down-regulation of SWI/SNF chromatin remodelling subunit-BRG1 and BRM were also frequently observed in HCC patients (Endo et al., 2013). In addition, the CHD family member Chromodomain helicase DNA binding 1 like (CHD1L) has been proven to have diverse oncogenic roles in hepatocarcinogenesis (Chen et al., 2010).

### Long non-coding RNAs (lncRNAs)

A large number of non-protein coding transcripts exist in the genome. In the past, those long non-coding RNAs were considered as “rubbish” of the genome for their unknown functions. Recently, emerging evidences suggested that lncRNAs might play important roles in regulating gene expression at post-transcriptional level. LncRNAs can regulate gene transcription either through directly binding to the RNA polymerase II, or modifying the activity of the transcriptional co-regulators (Mercer et al., 2009). In addition to transcriptional regulation, lncRNAs can also control the post-transcriptional mRNA processing such as mRNA splicing and translation. Furthermore, lncRNAs were also reported to be involved in regulating histone methylation and chromatin remodeling, which are the most important epigenetic regulatory machinery in regulating gene expression (Guttman and Rinn 2012). Altered expression of lncRNAs has been observed in tumors including HCC and they are suggested to play critical roles during tumorigenesis. High expression of lncRNA-HEIH is significantly associated with HCC recurrence and poor prognosis. *In vitro* and *in vivo* functional studies revealed that the overexpression of lncRNA-HEIH can promote HCC tumorigenesis and might function through EZH2 (Yang et al., 2011a). In addition, overexpression of Long Non-coding RNA HOTAIR and MALAT-1 could help predict tumor recurrence and prognosis of HCC patients (Lai et al., 2012; Yang et al., 2011b). All these evidences indicated that lncRNAs might be important in HCC initiation and progression.

## Summary and perspectives

**Figure 2:**
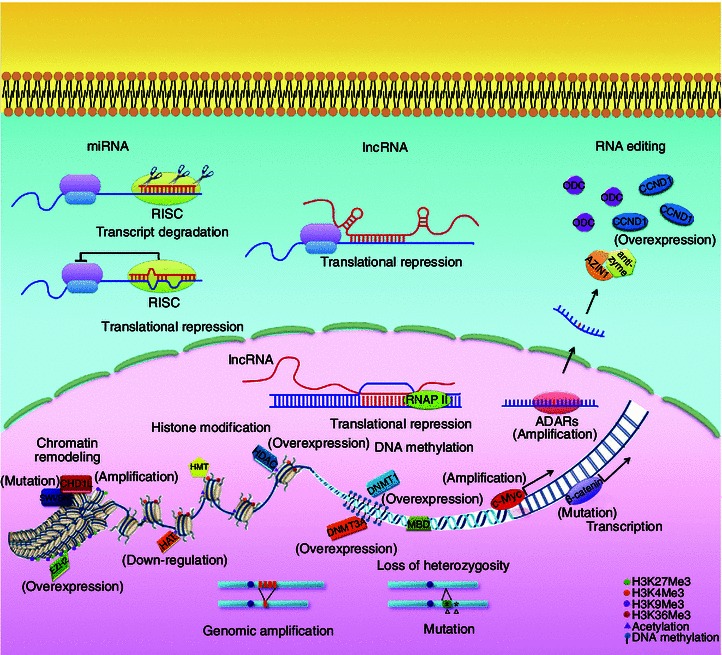
Genetic and epigenetic alterations in HCC. Genetic and epigenetic alterations interplay during cancer initiation and progression. The alterations exist at multiple levels including large chromosomal gain or loss, single nucleotide variations or mutations, overexpression or down-regulation of miRNAs and lncRNAs, disrupted RNA editing events, hyperactivation or inactivation of chromatin remodeling components, and aberrant DNA methylation at the promoter region of critical tumor suppressor genes et al

Like other solid tumors, HCC is characterized with multiple hallmarks including sustained proliferation, evading growth suppressive signals, metastasis to other organs, promoting angiogenesis, tumor-promoting microenvironment, cancer stem cell properties, and energy metabolism switch, etc. Genetic and epigenetic alterations interplay during cancer initiation and progression. The genomic changes vary from large chromosomal gain or loss to single nucleotide variations or mutations. Genetic alterations are irreversible alterations, which could be observed as early as in the pre-neoplastic stages. The early onset of genetic alterations indicated that they might be the tumor initiating steps in the development of cancer. Chromosome instability is the most common type of genetic alteration. Chromosome 1q and 8q are frequently amplified, while chromosome 1p, 4q, 6q, 9p, 16p, 16q, and 17p are frequently lost in HCC. Those hot regions usually harbor important oncogenes or tumor suppressor genes, which might significantly contribute to hepatocarcinogenesis. In addition to large chromosomal segmental changes, single nucleotide changes in the genome also make a substantial contribution to cancer progression. Nucleotide changes known as mutations or variations can lead to either gain-of-function or loss-of-function of oncogenes and tumor suppressor genes. Non-coding nucleotide changes can also affect gene transcription, and post-transcriptional regulations of critical tumor related genes, which may directly trigger oncogenesis or enhance cancer risk. Epigenetic alteration is another important mechanism for oncogenesis. Epigenetic regulation includes a wide range of regulations at transcriptional or post-transcriptional levels, such as DNA methylation, histone modification, chromatin remodeling, and lncRNAs. Alterations at the epigenetic regulation machinery may lead to disrupted gene expression, which can also cause the activation of oncogenes or inactivation of tumor suppressor genes. The genetic and epigenetic alterations in HCC are summarized in Fig. 2. Small molecules or monoclonal antibodies, which specifically target the altered onco-proteins, have already been proven to be efficient in treating several types of cancer. For example, imatinib, which specifically target the BCR-ABL fusion kinase, is used in treating chronic myeloid leukemia; transtuzumab, a monoclonal antibody targeting the amplified tyrosine kinase receptor HER2, is used to treat advanced-stage breast cancer. However, the targeted therapies which are effective in treating HCC are still limited. Better understanding and characterization of novel genetic and epigenetic alterations, which are important to hepatocarcinogenesis, may help understand the molecular pathogenesis of HCC, as well as providing novel therapeutic targets for HCC treatment.

## Abbreviations

Ang2: angiopoietin-2
bFGF: basic fibroblast growth factor
CGH: comparative genomic hybridization
CSC: cancer stem cell
ECM: extracellular matrix
GWAS: genome-wide association study
HCC: hepatocellular carcinoma
HGF: hepatoctye growth factor
lncRNAs: long non-coding RNAs
LOH: loss of heterozygosity
MMP: matrix metalloproteinases
PDGF: platelet-derived growth factor
RIZ1: Rb-interacting zinc finger 1
TAM: tumor associated macrophage
TCA: tricarboxylic acid

## References

[CR1] Adams JM, Cory S (2007). The Bcl-2 apoptotic switch in cancer development and therapy. Oncogene.

[CR2] Antoniou AC, Beesley J, McGuffog L, Sinilnikova OM, Healey S, Neuhausen SL, Ding YC, Rebbeck TR, Weitzel JN, Lynch HT (2010). Common breast cancer susceptibility alleles and the risk of breast cancer for BRCA1 and BRCA2 mutation carriers: implications for risk prediction. Cancer Res.

[CR3] Arii S, Mise M, Harada M, Furutani M, Ishigami S, Niwano M, Mizumoto M, Fukumoto M, Imamura M (1996). Overexpression of matrix metalloproteinase 9 gene in hepatocellular carcinoma with invasive potential. Hepatology.

[CR4] Armeanu S, Pathil A, Venturelli S, Mascagni P, Weiss TS, Gottlicher M, Gregor M, Lauer UM, Bitzer M (2005). Apoptosis on hepatoma cells but not on primary hepatocytes by histone deacetylase inhibitors valproate and ITF2357. J Hepatol.

[CR5] Athanasiadis A, Rich A, Maas S (2004). Widespread A-to-I RNA editing of Alu-containing mRNAs in the human transcriptome. PLoS Biol.

[CR6] Ben-Porath I, Thomson MW, Carey VJ, Ge R, Bell GW, Regev A, Weinberg RA (2008). An embryonic stem cell-like gene expression signature in poorly differentiated aggressive human tumors. Nat Genet.

[CR7] Beyoglu D, Imbeaud S, Maurhofer O, Bioulac-Sage P, Zucman-Rossi J, Dufour JF, Idle JR (2013). Tissue metabolomics of hepatocellular carcinoma: tumor energy metabolism and the role of transcriptomic classification. Hepatology.

[CR8] Bingle L, Brown NJ, Lewis CE (2002). The role of tumour-associated macrophages in tumour progression: implications for new anticancer therapies. J Pathol.

[CR9] Budhu A, Jia HL, Forgues M, Liu CG, Goldstein D, Lam A, Zanetti KA, Ye QH, Qin LX, Croce CM (2008). Identification of metastasis-related microRNAs in hepatocellular carcinoma. Hepatology.

[CR10] Cairns RA, Harris IS, Mak TW (2011). Regulation of cancer cell metabolism. Nat Rev Cancer.

[CR11] Calvisi DF, Ladu S, Gorden A, Farina M, Lee JS, Conner EA, Schroeder I, Factor VM, Thorgeirsson SS (2007). Mechanistic and prognostic significance of aberrant methylation in the molecular pathogenesis of human hepatocellular carcinoma. J Clin Invest.

[CR12] Carmeliet P, Jain RK (2000). Angiogenesis in cancer and other diseases. Nature.

[CR13] Cavallaro U, Christofori G (2004). Cell adhesion and signalling by cadherins and Ig-CAMs in cancer. Nat Rev Cancer.

[CR14] Chan THM, Chen LL, Liu M, Hu L, Zheng BJ, Poon VKM, Huang PZ, Yuan YF, Huang JD, Yang J (2012). Translationally controlled tumor protein induces mitotic defects and chromosome missegregation in hepatocellular carcinoma development. Hepatology.

[CR15] Chan TH, Lin CH, Qi L, Fei J, Li Y, Yong KJ, Liu M, Song Y, Chow RK, Ng VH (2013). A disrupted RNA editing balance mediated by ADARs (adenosine deaminases that act on RNA) in human hepatocellular carcinoma. Gut.

[CR16] Chang Y, Yan W, He XX, Zhang LM, Li CJ, Huang H, Nace G, Geller DA, Lin JS, Tsung A (2012). miR-375 inhibits autophagy and reduces viability of hepatocellular carcinoma cells under hypoxic conditions. Gastroenterology.

[CR17] Chen YC, Lin MC, Yao H, Wang H, Zhang AQ, Yu J, Hui CK, Lau GK, He ML, Sung J (2007). Lentivirus-mediated RNA interference targeting enhancer of zeste homolog 2 inhibits hepatocellular carcinoma growth through down-regulation of stathmin (vol. 46, p. 200, 2007). Hepatology.

[CR18] Chen LL, Hu L, Chan THM, Tsao GSW, Xie D, Huo KK, Fu L, Ma S, Zheng BJ, Guan XY (2009). Chromodomain helicase/adenosine triphosphatase DNA binding protein 1-like (CHD1L) gene suppresses the nucleus-to-mitochondria translocation of Nur77 to sustain hepatocellular carcinoma cell survival. Hepatology.

[CR19] Chen MH, Huang JD, Hu L, Zheng BJ, Chen LL, Tsang SL, Guan XY (2009). Transgenic CHD1L expression in mouse induces spontaneous tumors. PLoS One.

[CR20] Chen LL, Chan THM, Yuan YF, Hu L, Huang J, Ma S, Wang J, Dong SS, Tang KH, Xie D (2010). CHD1L promotes hepatocellular carcinoma progression and metastasis in mice and is associated with these processes in human patients. J Clin Invest.

[CR21] Chen LL, Yuan YF, Li Y, Chan THM, Zheng BJ, Huang J, Guan XY (2011). Clinical significance of CHD1L in hepatocellular carcinoma and therapeutic potentials of virus-mediated CHD1L depletion. Gut.

[CR22] Chen LL, Li Y, Lin CH, Chan THM, Chow RKK, Song YY, Liu M, Yuan YF, Fu L, Kong KL (2013). Recoding RNA editing of AZIN1 predisposes to hepatocellular carcinoma. Nat Med.

[CR189] Chochi Y, Kawauchi S, Nakao M, Furuya T, Hashimoto K, Oga A, Oka M, Sasaki K (2009) A copy number gain of the 6p arm is linked with advanced hepatocellular carcinoma: an array-based comparative genomic hybridization study. J Pathol 217:677–684 10.1002/path.2491 19097070

[CR23] Colombino M, Sperlongano P, Izzo F, Tatangelo F, Botti G, Lombardi A, Accardo M, Tarantino L, Sordelli I, Agresti M (2012). BRAF and PIK3CA genes are somatically mutated in hepatocellular carcinoma among patients from South Italy. Cell Death Dis.

[CR24] Datta J, Kutay H, Nasser MW, Nuovo GJ, Wang B, Majumder S, Liu CG, Volinia S, Croce CM, Schmittgen TD (2008). Methylation mediated silencing of microRNA-1 gene and its role in hepatocellular carcinogenesis. Cancer Res.

[CR25] Ding T, Xu J, Wang F, Shi M, Zhang Y, Li SP, Zheng LM (2009). High tumor-infiltrating macrophage density predicts poor prognosis in patients with primary hepatocellular carcinoma after resection. Hum Pathol.

[CR26] Ding J, Huang SL, Wu SQ, Zhao YJ, Liang LH, Yan MX, Ge C, Yao J, Chen TY, Wan DF (2010). Gain of miR-151 on chromosome 8q24.3 facilitates tumour cell migration and spreading through downregulating RhoGDIA. Nat Cell Biol.

[CR27] Ding J, Huang SL, Wang Y, Tian Q, Zha RP, Shi HB, Wang QF, Ge C, Chen TY, Zhao YJ (2013). Genome-wide screening reveals that miR-195 targets the TNF-/NF-B pathway by down-regulating IB kinase alpha and TAB3 in hepatocellular carcinoma. Hepatology.

[CR28] El Tayebi HM, Omar K, Hegy S, El Maghrabi M, El Brolosy M, Hosny KA, Esmat G, Abdelaziz AI (2013). Repression of miR-17-5p with elevated expression of E2F-1 and c-MYC in non-metastatic hepatocellular carcinoma and enhancement of cell growth upon reversing this expression pattern. Biochem Biophys Res Commun.

[CR29] El-Serag HB, Rudolph KL (2007). Hepatocellular carcinoma: epidemiology and molecular carcinogenesis. Gastroenterology.

[CR30] Endo M, Yasui K, Zen Y, Gen Y, Zen K, Tsuji K, Dohi O, Mitsuyoshi H, Tanaka S, Taniwaki M (2013). Alterations of the SWI/SNF chromatin remodelling subunit-BRG1 and BRM in hepatocellular carcinoma. Liver Int.

[CR185] Fang W, Piao Z, Simon D, Sheu JC, Huang S (2000) Mapping of a minimal deleted region in human hepatocellular carcinoma to 1p36.13–p36.23 and mutational analysis of the RIZ (PRDM2) gene localized to the region. Genes chromosomes cancer 28:269–275 10862032

[CR31] Farber E (1984). The multistep nature of cancer development. Cancer Res.

[CR32] Fattovich G, Giustina G, Degos F, Tremolada F, Diodati G, Almasio P, Nevens F, Solinas A, Mura D, Brouwer JT (1997). Morbidity and mortality in compensated cirrhosis type C: a retrospective follow-up study of 384 patients. Gastroenterology.

[CR33] Finn RS, Zhu AX (2009). Targeting angiogenesis in hepatocellular carcinoma: focus on VEGF and bevacizumab. Expert Rev Anticancer Ther.

[CR34] Fiume L, Manerba M, Vettraino M, Di Stefano G (2011). Effect of sorafenib on the energy metabolism of hepatocellular carcinoma cells. Eur J Pharmacol.

[CR35] Fodde R, Brabletz T (2007). Wnt/beta-catenin signaling in cancer stemness and malignant behavior. Curr Opin Cell Biol.

[CR36] Fornari F, Gramantieri L, Ferracin M, Veronese A, Sabbioni S, Calin GA, Grazi GL, Giovannini C, Croce CM, Bolondi L (2008). MiR-221 controls CDKN1C/p57 and CDKN1B/p27 expression in human hepatocellular carcinoma. Oncogene.

[CR37] Fornari F, Milazzo M, Chieco P, Negrini M, Calin GA, Grazi GL, Pollutri D, Croce CM, Bolondi L, Gramantieri L (2010). MiR-199a-3p Regulates mTOR and c-Met to influence the doxorubicin sensitivity of human hepatocarcinoma cells. Cancer Res.

[CR38] Fu L, Dong SS, Xie YW, Tai LS, Chen L, Kong KL, Man K, Xie D, Li Y, Cheng Y (2010). Down-regulation of tyrosine aminotransferase at a frequently deleted region 16q22 contributes to the pathogenesis of hepatocellular carcinoma. Hepatology.

[CR39] Fujimoto A, Totoki Y, Abe T, Boroevich KA, Hosoda F, Nguyen HH, Aoki M, Hosono N, Kubo M, Miya F (2012). Whole-genome sequencing of liver cancers identifies etiological influences on mutation patterns and recurrent mutations in chromatin regulators. Nat Genet.

[CR192] Fujiwara Y, Hoon DS, Yamada T, Umeshita K, Gotoh M, Sakon M, Nishisho I, Monden M (2000) PTEN / MMAC1 mutation and frequent loss of heterozygosity identified in chromosome 10q in a subset of hepatocellular carcinomas. JJpn J Cancer Res 91:287–292 10.1111/j.1349-7006.2000.tb00943.x PMC5926370 10760687

[CR40] Giannelli G, Bergamini C, Marinosci F, Fransvea E, Quaranta M, Lupo L, Schiraldi O, Antonaci S (2002). Clinical role of MMP-2/TIMP-2 imbalance in hepatocellular carcinoma. Int J Cancer.

[CR41] Gott JM, Emeson RB (2000). Functions and mechanisms of RNA editing. Annu Rev Genet.

[CR42] Gramantieri L, Ferracin M, Fornari F, Veronese A, Sabbioni S, Liu CG, Calin GA, Giovannini C, Ferrazzi E, Grazi GL (2007). Cyclin g1 is a target of miR-122a, a microRNA frequently down-regulated in human hepatocellular carcinoma. Cancer Res.

[CR43] Gramantieri L, Fornari F, Ferracin M, Veronese A, Sabbioni S, Calin GA, Grazi GL, Croce CM, Bolondi L, Negrini M (2009). MicroRNA-221 targets Bmf in hepatocellular carcinoma and correlates with tumor multifocality. Clin Cancer Res.

[CR44] Guan XY, Fang Y, Sham JST, Kwong DLW, Zhang YQ, Liang QW, Li HM, Zhou H, Trent JM (2000). Recurrent chromosome alterations in hepatocellular carcinoma detected by comparative genomic hybridization. Genes Chromosomes Cancer.

[CR45] Guichard C, Amaddeo G, Imbeaud S, Ladeiro Y, Pelletier L, Ben Maad I, Calderaro J, Bioulac-Sage P, Letexier M, Degos F (2012). Integrated analysis of somatic mutations and focal copy-number changes identifies key genes and pathways in hepatocellular carcinoma. Nat Genet.

[CR46] Guttman M, Rinn JL (2012). Modular regulatory principles of large non-coding RNAs. Nature.

[CR47] Hagiwara A, Cornu M, Cybulski N, Polak P, Betz C, Trapani F, Terracciano L, Heim MH, Ruegg MA, Hall MN (2012). Hepatic mTORC2 activates glycolysis and lipogenesis through Akt, glucokinase, and SREBP1c. Cell Metab.

[CR48] Hanahan D, Weinberg RA (2000). The hallmarks of cancer. Cell.

[CR49] Hanahan D, Weinberg RA (2011). Hallmarks of cancer: the next generation. Cell.

[CR50] He XX, Chang Y, Meng FY, Wang MY, Xie QH, Tang F, Li PY, Song YH, Lin JS (2012). MicroRNA-375 targets AEG-1 in hepatocellular carcinoma and suppresses liver cancer cell growth in vitro and in vivo. Oncogene.

[CR51] Hendrich B, Bird A (1998). Identification and characterization of a family of mammalian methyl-CpG binding proteins. Mol Cell Biol.

[CR52] Hengartner MO (2000). The biochemistry of apoptosis. Nature.

[CR53] Henry JC, Park JK, Jiang JM, Kim JH, Nagorney DM, Roberts LR, Banerjee S, Schmittgen TD (2010). miR-199a-3p targets CD44 and reduces proliferation of CD44 positive hepatocellular carcinoma cell lines. Biochem Biophys Res Commun.

[CR54] Hernandez-Gea V, Toffanin S, Friedman SL, Llovet JM (2013). Role of the microenvironment in the pathogenesis and treatment of hepatocellular carcinoma. Gastroenterology.

[CR250] Higashitsuji H, Itoh K, Nagao T, Dawson S, Nonoguchi K, Kido T, Mayer RJ, Arii S, Fujita J. (2000). Reduced stability of retinoblastoma protein by gankyrin, an oncogenic ankyrinrepeat protein overexpressed in hepatomas. Nat Med 6(1):96–99 10.1038/71600 10613832

[CR55] Hsu PP, Sabatini DM (2008). Cancer cell metabolism: Warburg and beyond. Cell.

[CR56] Hu L, Liu M, Chen LL, Chan THM, Wang J, Huo KK, Zheng BJ, Xie D, Guan XY (2012). SCYL1 binding protein 1 promotes the ubiquitin-dependent degradation of Pirh2 and has tumor-suppressive function in the development of hepatocellular carcinoma. Carcinogenesis.

[CR57] Hussain SP, Schwank J, Staib F, Wang XW, Harris CC (2007). TP53 mutations and hepatocellular carcinoma: insights into the etiology and pathogenesis of liver cancer. Oncogene.

[CR58] Iwata N, Yamamoto H, Sasaki S, Itoh F, Suzuki H, Kikuchi T, Kaneto H, Iku S, Ozeki I, Karino Y (2000). Frequent hypermethylation of CpG islands and loss of expression of the 14-3-3 sigma gene in human hepatocellular carcinoma. Oncogene.

[CR59] Jablkowski M, Bocian A, Bialkowska J, Bartkowiak J (2005). A comparative study of P53/MDM2 genes alterations and P53/MDM2 proteins immunoreactivity in liver cirrhosis and hepatocellular carcinoma. J Exp Clin Cancer Res.

[CR60] Ji JF, Yamashita T, Budhu A, Forgues M, Jia HL, Li CL, Deng CX, Wauthier E, Reid LM, Ye QH (2009). Identification of microRNA-181 by genome-wide screening as a critical player in EpCAM-positive hepatic cancer stem cells. Hepatology.

[CR61] Ji J, Zhao L, Budhu A, Forgues M, Jia HL, Qin LX, Ye QH, Yu J, Shi X, Tang ZY (2010). Let-7g targets collagen type I alpha2 and inhibits cell migration in hepatocellular carcinoma. J Hepatol.

[CR62] Jiang DK, Sun JL, Cao GW, Liu Y, Lin DX, Gao YZ, Ren WH, Long XD, Zhang HX, Ma XP (2013). Genetic variants in STAT4 and HLA-DQ genes confer risk of hepatitis B virus-related hepatocellular carcinoma. Nat Genet.

[CR63] Joyce JA (2005). Therapeutic targeting of the tumor microenvironment. Cancer Cell.

[CR64] Kanai Y, Ushijima S, Hui AM, Ochiai A, Tsuda H, Sakamoto M, Hirohashi S (1997). The E-cadherin gene is silenced by CpG methylation in human hepatocellular carcinomas. Int J Cancer.

[CR200] Kanai Y, Hui AM, Sun L, Ushijima S, Sakamoto M, Tsuda H, Hirohashi S (1999) DNA hypermethylation at the D17S5 locus and reduced HIC-1 mRNA expression are associated with hepatocarcinogenesis. Hepatology 29:703–709 10.1002/hep.510290338 10051471

[CR65] Kim KW, Bae SK, Lee OH, Bae MH, Lee MJ, Park BC (1998). Insulin-like growth factor II induced by hypoxia may contribute to angiogenesis of human hepatocellular carcinoma. Cancer Res.

[CR183] Kim TM, Yim SH, Shin SH, Xu HD, Jung YC, Park CK, Choi JY, Park WS, Kwon MS, Fiegler H et al (2008) Clinical implication of recurrent copy number alterations in hepatocellular carcinoma and putative oncogenes in recurrent gains on 1q. Int J Cancer 123:2808–2815. 10.1002/ijc.23901 PMC2698448 18803288

[CR66] Kim J, Woo AJ, Chu JL, Snow JW, Fujiwara Y, Kim CG, Cantor AB, Orkin SH (2010). A Myc network accounts for similarities between embryonic stem and cancer cell transcription programs. Cell.

[CR199] Ko E, Kim SJ, Joh JW, Park CK, Park J, Kim DH (2008) CpG island hypermethylation of SOCS-1 gene is inversely associated with HBV infection in hepatocellular carcinoma. Cancer Lett 271:240–250 10.1016/j.canlet.2008.06.009 18639978

[CR67] Kondo Y, Kanai Y, Sakamoto M, Mizokami M, Ueda R, Hirohashi S (2000). Genetic instability and aberrant DNA methylation in chronic hepatitis and cirrhosis - A comprehensive study of loss of heterozygosity and microsatellite instability at 39 loci and DNA hypermethylation on 8 CpG islands in microdissected specimens from patients with hepatocellular carcinoma. Hepatology.

[CR68] Kouzarides T (2007). Chromatin modifications and their function. Cell.

[CR69] Kroemer G, Pouyssegur J (2008). Tumor cell metabolism: cancer’s Achilles’ heel. Cancer Cell.

[CR70] Kubbutat MHG, Jones SN, Vousden KH (1997). Regulation of p53 stability by Mdm2. Nature.

[CR71] Kumar V, Kato N, Urabe Y, Takahashi A, Muroyama R, Hosono N, Otsuka M, Tateishi R, Omata M, Nakagawa H (2011). Genome-wide association study identifies a susceptibility locus for HCV-induced hepatocellular carcinoma. Nat Genet.

[CR195] Kuroki T, Fujiwara Y, Nakamori S, Imaoka S, Kanematsu T, Nakamura Y (1995) Evidence for the presence of two tumour-suppressor genes for hepatocellular carcinoma on chromosome 13q. Br J Cancer 72:383–385 10.1038/bjc.1995.342 PMC2034007 7640222

[CR72] Lai MC, Yang Z, Zhou L, Zhu QQ, Xie HY, Zhang F, Wu LM, Chen LM, Zheng SS (2012). Long non-coding RNA MALAT-1 overexpression predicts tumor recurrence of hepatocellular carcinoma after liver transplantation. Med Oncol.

[CR73] Lang QB, Ling CQ (2012). MiR-124 suppresses cell proliferation in hepatocellular carcinoma by targeting PIK3CA. Biochem Biophys Res Commun.

[CR74] Law PTY, Ching AKK, Chan AWH, Wong QWL, Wong CK, To KF, Wong N (2012). MiR-145 modulates multiple components of the insulin-like growth factor pathway in hepatocellular carcinoma. Carcinogenesis.

[CR75] Lee S, Lee HJ, Kim JH, Lee HS, Jang JJ, Kang GH (2003). Aberrant CpG island hypermethylation along multistep hepatocarcinogenesis. Am J Pathol.

[CR76] Lee TK, Poon RTP, Yuen AP, Ling MT, Kwok WK, Wang XH, Wong YC, Guan XY, Man K, Chau KL (2006). Twist overexpression correlates with hepatocellular carcinoma metastasis through induction of epithelial-mesenchymal transition. Clin Cancer Res.

[CR77] Lee TK, Castilho A, Cheung VC, Tang KH, Ma S, Ng IO (2011). CD24(+) liver tumor-initiating cells drive self-renewal and tumor initiation through STAT3-mediated NANOG regulation. Cell Stem Cell.

[CR78] Lemmon MA, Schlessinger J (2010). Cell signaling by receptor tyrosine kinases. Cell.

[CR79] Levine AJ, Puzio-Kuter AM (2010). The control of the metabolic switch in cancers by oncogenes and tumor suppressor genes. Science.

[CR80] Li N, Fu H, Tie Y, Hu Z, Kong W, Wu Y, Zheng X (2009). miR-34a inhibits migration and invasion by down-regulation of c-Met expression in human hepatocellular carcinoma cells. Cancer Lett.

[CR81] Li J, Fu H, Xu C, Tie Y, Xing R, Zhu J, Qin Y, Sun Z, Zheng X (2010). miR-183 inhibits TGF-beta1-induced apoptosis by downregulation of PDCD4 expression in human hepatocellular carcinoma cells. BMC Cancer.

[CR82] Li M, Zhao H, Zhang X, Wood LD, Anders RA, Choti MA, Pawlik TM, Daniel HD, Kannangai R, Offerhaus GJ (2011). Inactivating mutations of the chromatin remodeling gene ARID2 in hepatocellular carcinoma. Nat Genet.

[CR83] Li SP, Qian J, Yang Y, Zhao WT, Dai JC, Bei JX, Foo JN, McLaren PJ, Li ZQ, Yang JM (2012). GWAS identifies novel susceptibility loci on 6p21.32 and 21q21.3 for hepatocellular carcinoma in chronic hepatitis B virus carriers. PLoS Genet.

[CR84] Li CF, Wang YZ, Wang SF, Wu B, Hao JL, Fan HX, Ju Y, Ding YP, Chen LZ, Chu XY (2013). Hepatitis B virus mRNA-mediated miR-122 inhibition upregulates PTTG1-binding protein, which promotes hepatocellular carcinoma tumor growth and cell invasion. J Virol.

[CR85] Li Y, Chen LL, Chan THM, Guan XY (2013). Hepatocellular carcinoma: transcriptome diversity regulated by RNA editing. Int J Biochem Cell B.

[CR198] Li J, Quan H, Liu Q, Si Z, He Z, Qi H (2013c) Alterations of axis inhibition protein 1 (AXIN1) in hepatitis B virus-related hepatocellular carcinoma and overexpression of AXIN1 induces apoptosis in hepatocellular cancer cells. Oncol Res 20:281–288 10.3727/096504013x13639794277608 23879168

[CR86] Liu WH, Yeh SOH, Lu CC, Yu SL, Chen HY, Lin CY, Chen DS, Chen PJ (2009). MicroRNA-18a prevents estrogen receptor-alpha expression, promoting proliferation of hepatocellular carcinoma cells. Gastroenterology.

[CR87] Liu AM, Poon RTP, Luk JM (2010). MicroRNA-375 targets Hippo-signaling effector YAP in liver cancer and inhibits tumor properties. Biochem Biophys Res Commun.

[CR88] Liu M, Chen LL, Chan THM, Wang J, Li Y, Li Y, Zeng TT, Yuan YF, Guan XY (2012). Serum and glucocorticoid kinase 3 at 8q13.1 promotes cell proliferation and survival in hepatocellular carcinoma. Hepatology.

[CR89] Liu L, Dai Y, Chen J, Zeng T, Li Y, Chen L, Zhu YH, Li J, Xie D, Yuan YF (2013). Maelstrom promotes hepatocellular carcinoma metastasis by inducing epithelial–mesenchymal transition via Akt/GSK-3beta/snail signaling. Hepatology.

[CR90] Liu M, Li Y, Chen L, Chan THM, Song Y, Fu L, Zeng TT, Dai YD, Zhu YH, Li Y (2014). Allele-specific imbalance of oxidative stress-induced growth inhibitor 1 associates with progression of hepatocellular carcinoma. Gastroenterology.

[CR92] Luedde T (2010). MicroRNA-151 and its hosting gene FAK (focal adhesion kinase) regulate tumor cell migration and spreading of hepatocellular carcinoma. Hepatology.

[CR93] Ma S, Chan KW, Hu L, Lee TK, Wo JY, Ng IO, Zheng BJ, Guan XY (2007). Identification and characterization of tumorigenic liver cancer stem/progenitor cells. Gastroenterology.

[CR94] Ma NF, Hu L, Fung JM, Xie D, Zheng BJ, Chen LL, Tang DJ, Fu L, Wu Z, Chen M (2008). Isolation and characterization of a novel oncogene, amplified in liver cancer 1, within a commonly amplified region at 1q21 in hepatocellular carcinoma. Hepatology.

[CR95] Ma S, Tang KH, Chan YP, Lee TK, Kwan PS, Castilho A, Ng I, Man K, Wong N, To KF (2010). miR-130b promotes CD133(+) liver tumor-initiating cell growth and self-renewal via tumor protein 53-induced nuclear protein 1. Cell Stem Cell.

[CR96] Marquardt JU, Raggi C, Andersen JB, Seo D, Avital I, Geller D, Lee YH, Kitade M, Holczbauer A, Gillen MC (2011). Human hepatic cancer stem cells are characterized by common stemness traits and diverse oncogenic pathways. Hepatology.

[CR97] Meng FY, Henson R, Wehbe-Janek H, Ghoshal K, Jacob ST, Patel T (2007). MicroRNA-21 regulates expression of the PTEN tumor suppressor gene in human hepatocellular cancer. Gastroenterology.

[CR98] Mercer TR, Dinger ME, Mattick JS (2009). Long non-coding RNAs: insights into functions. Nat Rev Genet.

[CR99] Merle P, De La Monte S, Kim M, Herrmann M, Tanaka S, Von dem Bussche A, Kew MC, Trepo C, Wands JR (2004). Functional consequences of frizzled-7 receptor overexpression in human hepatocellular carcinoma. Gastroenterology.

[CR100] Mise M, Arii S, Higashituji H, Furutani M, Niwano M, Harada T, Ishigami SI, Toda Y, Nakayama H, Fukumoto M (1996). Clinical significance of vascular endothelial growth factor and basic fibroblast growth factor gene expression in liver turner. Hepatology.

[CR101] Mishra L, Banker T, Murray J, Byers S, Thenappan A, He AR, Shetty K, Johnson L, Reddy EP (2009). Liver stem cells and hepatocellular carcinoma. Hepatology.

[CR187] Miyoshi Y, Iwao K, Nagasawa Y, Aihara T, Sasaki Y, Imaoka S, Murata M, Shimano T, Nakamura Y (1998) Activation of the beta-catenin gene in primary hepatocellular carcinomas by somatic alterations involving exon 3. Cancer Res 58:2524–2527 9635572

[CR102] Mizuno S, Chijiwa T, Okamura T, Akashi K, Fukumaki Y, Niho Y, Sasaki H (2001). Expression of DNA methyltransferases DNMT1, 3A, and 3B in normal hematopoiesis and in acute and chronic myelogenous leukemia. Blood.

[CR103] Momparler RL (2003). Cancer epigenetics. Oncogene.

[CR104] Murakami Y, Tamori A, Itami S, Tanahashi T, Toyoda H, Tanaka M, Wu WH, Brojigin N, Kaneoka Y, Maeda A (2013). The expression level of miR-18b in hepatocellular carcinoma is associated with the grade of malignancy and prognosis. BMC Cancer.

[CR105] Nishida N, Fukuda Y, Kokuryu H, Toguchida J, Yandell DW, Ikenega M, Imura H, Ishizaki K (1993). Role and mutational heterogeneity of the P53-gene in hepatocellular-carcinoma. Cancer Res.

[CR193] Nishida N, Fukuda Y, Komeda T, Kita R, Sando T, Furukawa M, Amenomori M, Shibagaki I, Nakao K, Ikenaga M et al (1994) Amplification and overexpression of the cyclin D1 gene in aggressive human hepatocellular carcinoma. Cancer Res 54:3107–3110 8205525

[CR184] Nishimura T, Nishida N, Itoh T, Komeda T, Fukuda Y, Ikai I, Yamaoka Y, Nakao K (2005) Discrete breakpoint mapping and shortest region of overlap of chromosome arm 1q gain and 1p loss in human hepatocellular carcinoma detected by semiquantitative microsatellite analysis. Genes Chromosomes Cancer 42:34–43 10.1002/gcc.20117 15495198

[CR106] Noh JH, Chang YG, Kim MG, Jung KH, Kim JK, Bae HJ, Eun JW, Shen Q, Kim SJ, Kwon SH (2013). MiR-145 functions as a tumor suppressor by directly targeting histone deacetylase 2 in liver cancer. Cancer Lett.

[CR107] Oh BK, Kim H, Park HJ, Shim YH, Choi J, Park C, Park YN (2007). DNA methyltransferase expression and DNA methylation in human hepatocellular carcinoma and their clinicopathological correlation. Int J Mol Med.

[CR190] Oka Y, Waterland RA, Killian JK, Nolan CM, Jang HS, Tohara K, Sakaguchi S, Yao T, Iwashita A, Yata Y et al (2002) M6P/IGF2R tumor suppressor gene mutated in hepatocellular carcinomas in Japan. Hepatology 35:1153–1163 10.1053/jhep.2002.32669 11981765

[CR108] Okamoto H, Yasui K, Zhao C, Arii S, Inazawa A (2003). PTK2 and EIF3S3 genes may be amplification targets at 8q23-q24 and are associated with large hepatocellular carcinomas. Hepatology.

[CR109] Okano M, Bell DW, Haber DA, Li E (1999). DNA methyltransferases Dnmt3a and Dnmt3b are essential for de novo methylation and mammalian development. Cell.

[CR110] Olivier M, Hollstein M, Hainaut P (2010). TP53 mutations in human cancers: origins, consequences, and clinical use. Cold Spring Harb Perspect Biol.

[CR111] Ozturk M (1991). P53 mutation in hepatocellular-carcinoma after aflatoxin exposure. Lancet.

[CR112] Parada LA, Hallen M, Tranberg KG, Hagerstrand I, Bondeson L, Mitelman F, Johansson B (1998). Frequent rearrangements of chromosomes 1, 7, and 8 in primary liver cancer. Genes Chromosomes Cancer.

[CR113] Parkin DM, Bray F, Ferlay J, Pisani P (2005). Global cancer statistics, 2002. CA Cancer J Clin.

[CR114] Pathil A, Armeanu S, Venturelli S, Mascagni P, Weiss TS, Gregor M, Lauer UM, Bitzer M (2006). HDAC inhibitor treatment of hepatoma cells induces both TRAIL-independent apoptosis and restoration of sensitivity to TRAIL. Hepatology.

[CR115] Patil MA, Zhang J, Ho C, Cheung ST, Fan ST, Chen X (2006). Hedgehog signaling in human hepatocellular carcinoma. Cancer Biol Ther.

[CR116] Pei DQ, Kang TB, Qi HX (2000). Cysteine array matrix metalloproteinase (CA-MMP)/MMP-23 is a type II transmembrane matrix metalloproteinase regulated by a single cleavage for both secretion and activation. J Biol Chem.

[CR117] Reya T, Morrison SJ, Clarke MF, Weissman IL (2001). Stem cells, cancer, and cancer stem cells. Nature.

[CR118] Rousseau B, Menard L, Haurie V, Taras D, Blanc JF, Moreau-Gaudry F, Metzler P, Hugues M, Boyault S, Lemiere S (2007). Overexpression and role of the ATPase and putative DNA helicase RuvB-like 2 in human hepatocellular carcinoma. Hepatology.

[CR119] Rundhaug JE (2003). Matrix metalloproteinases, angiogenesis, and cancer—Commentary re: A. C. Lockhart et al., reduction of wound angiogenesis in patients treated with BMS-275291, a broad spectrum matrix metalloproteinase inhibitor. Clin Cancer Res.

[CR120] Saito Y, Kanai Y, Nakagawa T, Sakamoto M, Saito H, Ishii H, Hirohashi S (2003). DNA methyltransferase (DNMT) 1 protein expression is significantly increased in human hepatocellular carcinomas with malignant potential and may be a biological predictor of prognosis in hepatocellular carcinoma patients. Hepatology.

[CR121] Santoni-Rugiu E, Jensen MR, Thorgeirsson SS (1998). Disruption of the pRb/E2F pathway and inhibition of apoptosis are major oncogenic events in liver constitutively expressing c-myc and transforming growth factor alpha. Cancer Res.

[CR122] Semela D, Dufour JF (2004). Angiogenesis and hepatocellular carcinoma. J Hepatol.

[CR123] Shachaf CM, Kopelman AM, Arvanitis C, Karlsson A, Beer S, Mandl S, Bachmann MH, Borowsky AD, Ruebner B, Cardiff RD (2004). MYC inactivation uncovers pluripotent differentiation and tumour dormancy in hepatocellular cancer. Nature.

[CR124] Shih TC, Tien YJ, Wen CJ, Yeh TS, Yu MC, Huang CH, Lee YS, Yen TC, Hsieh SY (2012). MicroRNA-214 downregulation contributes to tumor angiogenesis by inducing secretion of the hepatoma-derived growth factor in human hepatoma. J Hepatol.

[CR125] Shiraki K, Sugimoto K, Fujikawa K, Yamanaka T, Takase K, Nakano T (2000). Survivin promotes cell proliferation in human hepatocellular carcinoma. Gastroenterology.

[CR126] Song K, Han C, Wu T (2012). Epigenetic regulation of miR-122 expression by PPAR gamma/RXR alpha complex and HBx in hepatocellular carcinoma. Hepatology.

[CR127] Stamenkovic I (2000). Matrix metalloproteinases in tumor invasion and metastasis. Semin Cancer Biol.

[CR128] Sudo T, Utsunomiya T, Mimori K, Nagahara H, Ogawa K, Inoue H, Wakiyama S, Fujita H, Shirouzu K, Mori M (2005). Clinicopathological significance of EZH2 mRNA expression in patients with hepatocellular carcinoma. Br J Cancer.

[CR129] Sugimachi K, Tanaka S, Kameyama T, Taguchi KI, Aishima SI, Shimada M, Sugimachi K, Tsuneyoshi M (2003). Transcriptional repressor snail and progression of human hepatocellular carcinoma. Clin Cancer Res.

[CR130] Takehara T, Liu X, Fujimoto J, Friedman SL, Takahashi H (2001). Expression and role of Bcl-xL in human hepatocellular carcinomas. Hepatology.

[CR131] Thorgeirsson SS, Grisham JW (2002). Molecular pathogenesis of human hepatocellular carcinoma. Nat Genet.

[CR194] Tsujiuchi T, Sugata E, Masaoka T, Onishi M, Fujii H, Shimizu K, Honoki K (2007) Expression and DNA methylation patterns of Tslc1 and Dal-1 genes in hepatocellular carcinomas induced by N-nitrosodiethylamine in rats. Cancer Sci 98:943–948 10.1111/j.1349-7006.2007.00480.x PMC11158029 17428255

[CR132] Ueki T, Fujimoto J, Suzuki T, Yamamoto H, Okamoto E (1997). Expression of hepatocyte growth factor and its receptor, the c-met proto-oncogene, in hepatocellular carcinoma. Hepatology.

[CR133] Venook AP, Papandreou C, Furuse J, de Guevara LL (2010). The incidence and epidemiology of hepatocellular carcinoma: a global and regional perspective. Oncologist.

[CR134] Vertino PM, Sekowski JA, Coll JM, Applegren N, Han S, Hickey RJ, Malkas LH (2002). DNMT1 is a component of a multiprotein DNA replication complex. Cell Cycle.

[CR135] Vogelstein B, Kinzler KW (1993). The multistep nature of cancer. Trends Genet.

[CR136] Voulgari A, Pintzas A (2009). Epithelial–mesenchymal transition in cancer metastasis: mechanisms, markers and strategies to overcome drug resistance in the clinic. BBA-Rev Cancer.

[CR191] Wang G, Huang CH, Zhao Y, Cai L, Wang Y, Xiu SJ, Jiang ZW, Yang S, Zhao T, Huang W et al (2000) Genetic aberration in primary hepatocellular carcinoma: correlation between p53 gene mutation and loss-of-heterozygosity on chromosome 16q21–q23 and 9p21–p23. Cell Res 10:311–323 10.1038/sj.cr.7290058 11191353

[CR137] Wang Y, Wu MC, Sham JST, Zhang WG, Wu WQ, Guan XY (2002). Prognostic significance of c-myc and AIB1 amplification in hepatocellular carcinoma—a broad survey using high-throughput tissue microarray. Cancer-Am Cancer Soc.

[CR138] Wang GG, Allis CD, Chi P (2007). Chromatin remodeling and cancer, part I: covalent histone modifications. Trends Mol Med.

[CR139] Wang GG, Allis CD, Chi P (2007). Chromatin remodeling and cancer, Part II: ATP-dependent chromatin remodeling. Trends Mol Med.

[CR140] Wang Y, Lee ATC, Ma JZI, Wang JB, Ren JW, Yang YC, Tantoso E, Li KB, Ooi LLPJ, Tan P (2008). Profiling microRNA expression in hepatocellular carcinoma reveals microRNA-224 up-regulation and apoptosis inhibitor-5 as a microRNA-224-specific target. J Biol Chem.

[CR141] Wang B et al (2010a) TGFbeta-mediated upregulation of hepatic miR-181b promotes hepatocarcinogenesis by targeting TIMP3. Oncogene 29:1787–1797 10.1038/onc.2009.468 PMC2845743 20023698

[CR142] Wang Y, Lu YW, Toh ST, Sung WK, Tan P, Chow P, Chung AYF, Jooi LLP, Lee CGL (2010). Lethal-7 is down-regulated by the hepatitis B virus x protein and targets signal transducer and activator of transcription 3. J Hepatol.

[CR143] Wang L, Guo ZY, Zhang R, Xin B, Chen R, Zhao J, Wang T, Wen WH, Jia LT, Yao LB (2013). Pseudogene OCT4-pg4 functions as a natural micro RNA sponge to regulate OCT4 expression by competing for miR-145 in hepatocellular carcinoma. Carcinogenesis.

[CR144] Wang R, Zhao N, Li S, Fang JH, Chen MX, Yang J, Jia WH, Yuan Y, Zhuang SM (2013). MicroRNA-195 suppresses angiogenesis and metastasis of hepatocellular carcinoma by inhibiting the expression of VEGF, VAV2, and CDC42. Hepatology.

[CR145] Wei Y, Van Nhieu JT, Prigent S, Srivatanakul P, Tiollais P, Buendia MA (2002). Altered expression of E-cadherin in hepatocellular carcinoma: correlations with genetic alterations, beta-catenin expression, and clinical features. Hepatology.

[CR146] Whiteside TL (2008). The tumor microenvironment and its role in promoting tumor growth. Oncogene.

[CR147] Wong CM, Lee JMF, Ching YP, Jin DY, Ng IOL (2003). Genetic and epigenetic alterations of DLC-1 gene in hepatocellular carcinoma. Cancer Res.

[CR148] Wong QWL, Lung RWM, Law PTY, Lai PBS, Chan KYY, To KF, Wong N (2008). MicroRNA-223 is commonly repressed in hepatocellular carcinoma and potentiates expression of Stathmin1. Gastroenterology.

[CR149] Wong CCL, Wong CM, Tung EKK, Au SLK, Lee JMF, Poon RTP, Man K, Ng IOL (2011). The MICRORNA miR-139 suppresses metastasis and progression of hepatocellular carcinoma by down-regulating rho-kinase 2. Gastroenterology.

[CR150] Xia HP, Ooi LLPJ, Hui KM (2012). MiR-214 Targets beta-catenin pathway to suppress invasion, stem-like traits and recurrence of human hepatocellular carcinoma. PLoS One.

[CR151] Xiong YJ et al (2010) Effects of microRNA-29 on apoptosis, tumorigenicity, and prognosis of hepatocellular carcinoma. Hepatology 51:836–845 10.1002/hep.23380 20041405

[CR152] Xu T, Zhu Y, Xiong Y, Ge YY, Yun JP, Zhuang SM (2009). MicroRNA-195 suppresses tumorigenicity and regulates G1/S transition of human hepatocellular carcinoma cells. Hepatology.

[CR153] Xu H, He JH, Xiao ZD, Zhang QQ, Chen YQ, Zhou H, Qu LH (2010). Liver-enriched transcription factors regulate microRNA-122 that targets CUTL1 during liver development. Hepatology.

[CR154] Xu X, Fan Z, Kang L, Han J, Jiang C, Zheng X, Zhu Z, Jiao H, Lin J, Jiang K (2013). Hepatitis B virus X protein represses miRNA-148a to enhance tumorigenesis. J Clin Invest.

[CR155] Yamashita T, Budhu A, Forgues M, Wang XW (2007). Activation of hepatic stem cell marker EpCAM by Wnt-beta-catenin signaling in hepatocellular carcinoma. Cancer Res.

[CR156] Yamashita T, Ji J, Budhu A, Forgues M, Yang W, Wang HY, Jia H, Ye Q, Qin LX, Wauthier E (2009). EpCAM-positive hepatocellular carcinoma cells are tumor-initiating cells with stem/progenitor cell features. Gastroenterology.

[CR157] Yancopoulos GD, Davis S, Gale NW, Rudge JS, Wiegand SJ, Holash J (2000). Vascular-specific growth factors and blood vessel formation. Nature.

[CR158] Yang B, Guo MZ, Herman JG, Clark DP (2003). Aberrant promoter methylation profiles of tumor suppressor genes in hepatocellular carcinoma. Am J Pathol.

[CR159] Yang ZF, Ho DW, Ng MN, Lau CK, Yu WC, Ngai P, Chu PW, Lam CT, Poon RT, Fan ST (2008). Significance of CD90+ cancer stem cells in human liver cancer. Cancer Cell.

[CR160] Yang F, Yin YX, Wang F, Wang YQ, Zhang L, Tang Y, Sun SH (2010). miR-17-5p promotes migration of human hepatocellular carcinoma cells through the P38 mitogen-activated protein kinase-heat shock protein 27 pathway. Hepatology.

[CR161] Yang F, Zhang L, Huo XS, Yuan JH, Xu D, Yuan SX, Zhu N, Zhou WP, Yang GS, Wang YZ (2011). Long noncoding RNA high expression in hepatocellular carcinoma facilitates tumor growth through enhancer of zeste homolog 2 in humans. Hepatology.

[CR162] Yang Z, Zhou L, Wu LM, Lai MC, Xie HY, Zhang F, Zheng SS (2011). Overexpression of long non-coding RNA HOTAIR predicts tumor recurrence in hepatocellular carcinoma patients following liver transplantation. Ann Surg Oncol.

[CR163] Yang X, Liang L, Zhang XF, Jia HL, Qin Y, Zhu XC, Gao XM, Qiao P, Zheng Y, Sheng YY (2013). MicroRNA-26a suppresses tumor growth and metastasis of human hepatocellular carcinoma by targeting interleukin-6-Stat3 pathway. Hepatology.

[CR164] Yao YJ, Ping XL, Zhang H, Chen FF, Lee PK, Ahsan H, Chen CJ, Lee PH, Pleacocke M, Santella RM (1999). PTEN/MMAC1 mutations in hepatocellular carcinomas. Oncogene.

[CR196] Yasui K, Arii S, Zhao C, Imoto I, Ueda M, Nagai H, Emi M, Inazawa J (2002) TFDP1, CUL4A, and CDC16 identified as targets for amplification at 13q34 in hepatocellular carcinomas. Hepatology 35:1476–1484 10.1053/jhep.2002.33683 12029633

[CR165] Yau WL, Lam CSC, Ng L, Chow AKM, Chan STC, Chan JYK, Wo JYH, Ng KTP, Man K, Poon RTP (2013). Over-expression of miR-106b promotes cell migration and metastasis in hepatocellular carcinoma by activating epithelial–mesenchymal transition process. PLoS One.

[CR166] Ye QH, Qin LX, Forgues M, He P, Kim JW, Peng AC, Simon R, Li Y, Robles AI, Chen YD (2003). Predicting hepatitis B virus-positive metastatic hepatocellular carcinomas using gene expression profiling and supervised machine learning. Nat Med.

[CR167] Ying Q, Liang L, Guo W, Zha R, Tian Q, Huang S, Yao J, Ding J, Bao M, Ge C (2011). Hypoxia-inducible microRNA-210 augments the metastatic potential of tumor cells by targeting vacuole membrane protein 1 in hepatocellular carcinoma. Hepatology.

[CR168] Yoshiji H, Kuriyama S, Yoshii J, Ikenaka Y, Noguchi R, Hicklin DJ, Huber J, Nakatani T, Tsujinoue H, Yanase K (2002). Synergistic effect of basic fibroblast growth factor and vascular endothelial growth factor in murine hepatocellular carcinoma. Hepatology.

[CR169] Yoshikawa H, Matsubara K, Qian GS, Jackson P, Groopman JD, Manning JE, Harris CC, Herman JG (2001). SOCS-1, a negative regulator of the JAK/STAT pathway, is silenced by methylation in human hepatocellular carcinoma and shows growth-suppression activity. Nat Genet.

[CR170] Yuan BZ, Miller MJ, Keck CL, Zimonjic DB, Thorgeirsson SS, Popescu NC (1998). Cloning, characterization, and chromosomal localization of a gene frequently deleted in human liver cancer (DLC-1) homologous to rat RhoGAP. Cancer Res.

[CR171] Yuan F, Zhou W, Zou C, Zhang Z, Hu H, Dai Z, Zhang Y (2010). Expression of Oct4 in HCC and modulation to wnt/beta-catenin and TGF-beta signal pathways. Mol Cell Biochem.

[CR172] Yuen MF, Hou JL, Chutaputti A (2009). Hepatocellular carcinoma in the Asia pacific region. J Gastroenterol Hepatol.

[CR173] Yuneva MO, Fan TWM, Allen TD, Higashi RM, Ferraris DV, Tsukamoto T, Mates JM, Alonso FJ, Wang CM, Seo Y (2012). The metabolic profile of tumors depends on both the responsible genetic lesion and tissue type. Cell Metab.

[CR174] Zhang XY, Liu SR, Hu TS, Liu SP, He Y, Sun SH (2009). Up-regulated MicroRNA-143 transcribed by nuclear factor kappa B enhances hepatocarcinoma metastasis by repressing fibronectin expression. Hepatology.

[CR186] Zhang YJ, Ahsan H, Chen Y, Lunn RM, Wang LY, Chen SY, Lee PH, Chen CJ, Santella RM (2002) High frequency of promoter hypermethylation of RASSF1A and p16 and its relationship to aflatoxin B1-DNA adduct levels in human hepatocellular carcinoma. Mol Carcinog 35:85–92 10.1002/mc.10076 12325038

[CR175] Zhang HX, Zhai Y, Hu ZB, Wu C, Qian J, Jia WH, Ma FC, Huang WF, Yu LX, Yue W (2010). Genome-wide association study identifies 1p36.22 as a new susceptibility locus for hepatocellular carcinoma in chronic hepatitis B virus carriers. Nat Genet.

[CR176] Zhang YZ, Takahashi S, Tasaka A, Yoshima T, Ochi H, Chayama K (2013). Involvement of microRNA-224 in cell proliferation, migration, invasion, and anti-apoptosis in hepatocellular carcinoma. J Gastroenterol Hepatol.

[CR201] Zhao XT, Li JJ, He YH, Lan F, Fu LL, Guo JY, Zhao RJ, Ye Y, He M, Chong WM et al (2001) A novel growth suppressor gene on chromosome 17p13.3 with a high frequency of mutation in human hepatocellular carcinoma. Cancer Res 61:7383–7387 11606366

[CR177] Zheng F, Liao YJ, Cai MY, Liu YH, Liu TH, Chen SP, Bian XW, Guan XY, Lin MC, Zeng YX (2012). The putative tumour suppressor microRNA-124 modulates hepatocellular carcinoma cell aggressiveness by repressing ROCK2 and EZH2. Gut.

[CR178] Zheng X, Gai XH, Ding FH, Lu ZT, Tu KS, Yao YM, Liu QG (2013). Histone acetyltransferase PCAF up-regulated cell apoptosis in hepatocellular carcinoma via acetylating histone H4 and inactivating AKT signaling. Mol Cancer.

[CR179] Zhou XL, Thorgeirsson SS, Popescu NC (2004). Restoration of DLC-1 gene expression induces apoptosis and inhibits both cell growth and tumorigenicity in human hepatocellular carcinoma cells. Oncogene.

[CR180] Zhou L, Yang ZX, Song WJ, Li QJ, Yang F, Wang DS, Zhang N, Dou KF (2013). MicroRNA-21 regulates the migration and invasion of a stem-like population in hepatocellular carcinoma. Int J Oncol.

[CR181] Zhu AX, Duda DG, Sahani DV, Jain RK (2011). HCC and angiogenesis: possible targets and future directions. Nat Rev Clin Oncol.

[CR182] Zhu Y, Lu Y, Zhang Q, Liu JJ, Li TJ, Yang JR, Zeng CX, Zhuang SM (2012). MicroRNA-26a/b and their host genes cooperate to inhibit the G1/S transition by activating the pRb protein. Nucleic Acids Res.

[CR188] Zondervan PE, Wink J, Alers JC, JN IJ, Schalm SW, de Man RA, van Dekken H (2000) Molecular cytogenetic evaluation of virus-associated and non-viral hepatocellular carcinoma: analysis of 26 carcinomas and 12 concurrent dysplasias. J Pathol 192:207–215 10.1002/1096-9896(2000)9999:9999<::AID-PATH690>3.0.CO;2-# 11004697

